# The Effect of the Tetraalkylammonium Cation in the Electrochemical CO_2_ Reduction Reaction on Copper Electrode

**DOI:** 10.1021/acscatal.4c02297

**Published:** 2024-08-14

**Authors:** Connor Deacon-Price, Louis Changeur, Cássia S. Santana, Amanda C. Garcia

**Affiliations:** Van’t Hoff Institute for Molecular Sciences, University of Amsterdam, Science Park 904, 1098 XH Amsterdam, The Netherlands

**Keywords:** organic solvent, cation effect, Cu electrodes, product distribution, dry solvent

## Abstract

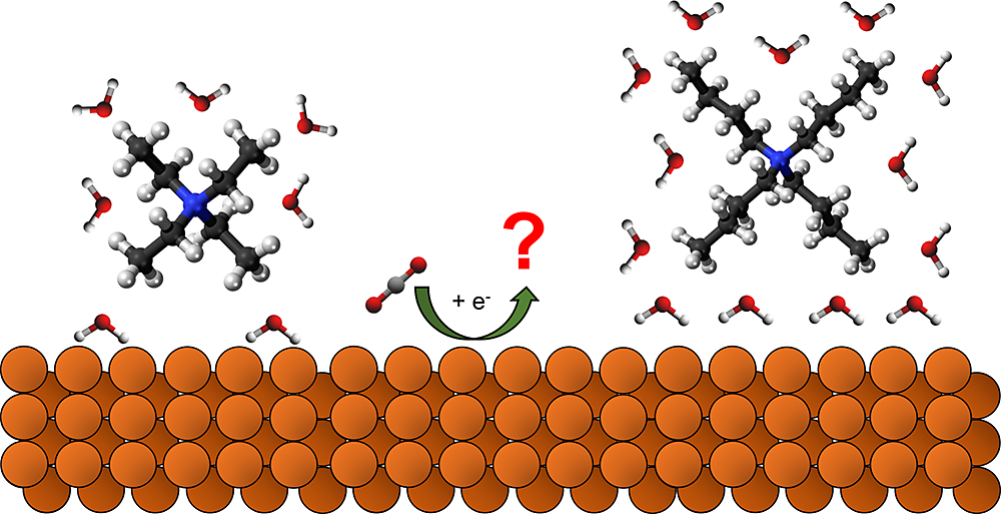

Aprotic organic solvents such as acetonitrile offer a potential solution to promote electrochemical CO_2_ reduction over the competing hydrogen evolution reaction. Tetraalkylammonium cations (TAA^+^) are widely used as supporting electrolytes in organic media due to their high solubility and conductivity. The alkyl chain length of TAA^+^ cations is known to influence electron transfer processes in electrochemical systems by the adsorption of TAA^+^, causing modifications of the double layer. In this work, we elucidate the influence of the cation chain length on the mechanism and selectivity of the CO_2_RR reaction under controlled dry and wet acetonitrile conditions on copper cathodes. We find that the hydrophobic hydration character of the cation, which can be tuned by the chain length, has an effect on product distribution, altering the reaction pathway. Under dry conditions, smaller cations (TEA^+^) preferentially promote oxalate production via dimerization of the CO_2_^·–^ intermediate, whereas formate is favored in the presence of water via protonation reaction. Larger cations (TBA^+^ > TPA^+^ > TEA^+^) favor the generation of CO regardless of water content. In situ FTIR analysis showed that TBA^+^ cations are able to stabilize adsorbed CO more effectively than TEA^+^, explaining why larger cations generate a higher proportion of CO. Our findings also suggest that higher cation concentrations suppress hydrogen evolution, particularly with larger cations, highlighting the role of cation chain length size and hydrophobic hydration shell.

## 1 Introduction

The electrochemical reduction of carbon dioxide (CO_2_RR) is an ongoing area of research with significant implications for sustainable energy conversion and environmental remediation.^1^· This process offers a promising avenue for the utilization of CO_2_, a prevalent greenhouse gas, while simultaneously producing valuable chemicals and fuels.^2−4^ Most of the studies in CO_2_RR are performed in aqueous electrolytes, and therefore the hydrogen evolution reaction (HER) competes with CO_2_RR, via either the direct reduction of protons (2H^+^ + e^–^ → H_2_) or the reduction of the solvent itself (2H_2_O + 2e^–^ → H_2_ + 2OH^–^), lowering the Faradaic efficiency of CO_2_RR products.^2−5^

Tailoring catalyst surface and electrolyte composition, and especially pH and supporting electrolyte cation, are the most common ways to steer activity and selectivity of CO_2_RR^6,7^ Murata et al., explored the impact of metal alkali cations on CO_2_RR. Their findings indicate that the hydrogen evolution reaction (HER) is favored by smaller Li^+^ cations, while larger cations, following the sequence Cs^+^ > K^+^ > Na^+^ > Li^+^, preferentially drive CO_2_ toward C_2+_ hydrocarbon products.^8^ Three main theories have been suggested to explain the role of the cation on the activity and selectivity of electrocatalytic processes.^9−11^ These are by the buffering of the interfacial pH at the interface, changes in the local electric field and stabilization of reaction intermediates, specifically the ability of the metal cation stabilizing the negatively charged reaction intermediate, *CO_2_^–^, a key intermediate in CO_2_RR to multicarbon products.^8,12^ Despite advancements, effectively controlling the competition between the CO_2_RR and HER remains an unresolved challenge.

In our latest study, we replaced the aqueous solvent with an aprotic one, namely acetonitrile, aiming to reduce hydrogen evolution during CO_2_RR on Cu-based electrodes.^13^ Our findings reveal that in acetonitrile, the presence of CO_2_ strongly inhibits HER by driving away water from the interface, even upon the addition of 1000 mM water into the solvent. Predominant CO production indicates that in acetonitrile, CO adsorption is inhibited compared to that of aqueous media.

While in aqueous media, the mechanism involved in the CO_2_RR is complex and involves multiple steps, which are either are or are not pH-dependent,^6,7,14^ in aprotic organic media, three major pathways are involved.^15^ According to Amatore and Savéant,^15^ the first step is similar to aqueous solutions, whereby an electron is transferred to a CO_2_ molecule, generating a CO_2_^·–^ radical 1), which is then followed by three competing pathways. A disproportionation reaction via reaction with CO_2_ molecule may occur, generating stoichiometric amounts of carbon monoxide (CO) and carbonate (CO_3_^2–^) 2), or a dimerization reaction with a second CO_2_^·–^ radical moiety to produce oxalate 3), bonding via C–O or C–C, respectively.^15^ In addition, CO_2_^·–^ may react with residual water in the electrolyte, producing formate 4).^16−18^
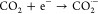
1
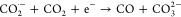
2
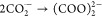
3
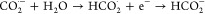
4

Currently, it is thought that the CO_2_^·–^ radicals are not exclusively localized at the cathode surface.^19,20^ Radicals react between the electrode surface and outer Helmholtz plane (OHP) with CO_2_ via a nucleophilic attack to form the C–O–C adduct which then further disproportionate to form CO and CO_3_^2–^.^15^ Oxalate, on the other hand, is believed to be formed via radical recombination in the diffusion-reaction layer outside of the influence of the electrostatics of the cathode. This is contrary to aqueous electrolytes, whereby CO_2_RR intermediates exist as adsorbates bound to the cathode surface.^21−23^ Despite this, whether the CO_2_^·–^ radical is formed through surface-bound intermediates or which parameters affect the reaction pathway in aprotic media is under debate.^24,25^ An alternate adsorption-based mechanism is provided (Scheme S1). Residual water content in the organic electrolyte has been identified as having a significant impact on the reaction pathway. Oxalate is favored in the absence of water, whereas in its presence, formate is produced.^17,25−31^

Unlike in aqueous media, where metal alkali cations typically serve as electrolytes, tetraalkylammonium cations (TAA^+^) are widely used as the supporting electrolyte in organic solvents due to their high solubility and are at the core of studies investigating the effect of hydrophobicity on ion hydration and ion–ion interactions in aqueous solutions.^32^ The hydrophobic character of TAA^+^ is considered to be tunable via the length of the four alkyl chains attached to the central nitrogen atom.

It is also reported that the size of the alkyl chain length of TAA^+^ cations influences electron transfer processes of electrochemical systems. This occurs by the adsorption of TAA^+^, causing modification of the electrode double layer.^33^

Therefore, in this work, we investigate the effect of tetraalkylammonium cation chain length in the selectivity of the CO_2_RR in nonaqueous aprotic media (acetonitrile). While a variety of conditions, including solvent, supporting electrolyte identity and concentration, and applied potentials, have been investigated previously,^17,25−31^ systematic studies are still lacking. In addition, the cation concentration and water content have also been investigated.

Through a combination of techniques including electrochemical measurements with online gas chromatography (GC) and in situ Fourier transform infrared (FTIR) spectroscopy, our study elucidates the significant influence of tetraalkylammonium cation size on the activity and selectivity of CO_2_RR in acetonitrile. Under dry acetonitrile conditions, we find that smaller cations (TEA^+^) preferentially promote oxalate production via the dimerization step (3), whereas the step to formate (4) is favored in the presence of water. Larger cations (TBA^+^ > TPA^+^ > TEA^+^) favor the generation of CO (2) regardless of water content. In situ FTIR shows that TBA^+^ cations are able to stabilize adsorbed CO more effectively than TEA^+^, explaining why larger cations generate a higher proportion of CO.

Our findings also suggest that higher cation concentrations suppress hydrogen evolution, particularly with larger cations, highlighting the role of cation chain length size and hydrophobic hydration shell. The exact reason why the mechanism for CO_2_RR changes as a function of the cation chain length is still not fully understood, and further investigation using in situ SFG (sum frequency generation spectroscopy) needs to be performed to evaluate the adsorption of cations and residual water on the surface of the electrode. Nonetheless, this work contributes to the understanding of cation influence in aprotic media, paving the way for a more targeted and efficient acetonitrile CO_2_RR system design.

## 2 Materials and Methods

### 2.1 Experimental Condition and Reactants

Ultrapure water (Milli-Q gradient, ≥18.2 MΩ cm, TOC 2.1 ppb) was used for all experiments in this work. Prior to each experiment, all glassware was cleaned by soaking overnight in a solution of 1 g/L KMnO_4_ (Fluka, ACS reagent) and 0.5 M H_2_SO_4_ (Sigma-Aldrich, 95–98%). Traces of KMnO_4_ and MnO_2_ were removed from the glassware by rinsing with ultrapure water, followed by immersion in a dilute H_2_SO_4_/H_2_O_2_ (1:0.3 M) solution. Afterward, the glassware was boiled in ultrapure water at least three times before using. When working in dry conditions, the one-compartment cell and the homemade PEEK H-cell and their associated components were put in an oven, at 130 °C, for at least 30 min to evaporate residual water.

Other chemicals used in this work were acetonitrile (MeCN) (ThermoFisher Scientific, 99.9%, extra dry over molecular sieve, AcroSeal) and tetraethylammonium tetrafluoroborate (TEATFB, ThermoFisher Scientific, 99%), tetrapropylammonium tetrafluoroborate (TPATFB, Sigma-Aldrich, ≥98%), tetra-*n*-butylammonium tetrafluoroborate (TBATFB, Alfa Aesar, 99%), and NaOH (MaTecK, p.a., ≥99.9%). It is important to note that all tetraalkylammonium cations used in this study contain the same BF4^–^ anion, as the borate anion can undergo hydrolysis in aqueous environments.^34^ Therefore, any observed differences in our results can be attributed to the different cations and not to the anions. Additionally, the water content was rigorously controlled to ensure consistent experimental conditions for each electrolyte used in this work. The solvent (MeCN) was used as received, whereas the salts TEATFB, TPATFB, and TBATFB were dried over P_2_O_5_. The procedure to dry the salts consisted of adding the necessary amount of salt in a Schlenk tube under vacuum, connected in series to another Schlenk tube containing solid P_2_O_5_. Water contained in the salt evaporates under a vacuum and reacts with P_2_O_5_ to form H_3_PO_4_. The salts were allowed to dry for at least 2 days before use in experiments. Further drying of electrolytes was performed with 3 Å molecular sieves which were flame-dried under vacuum. After the preparation of stock solutions of the organic electrolytes, their content of water was measured by Karl Fischer titration. CO_2_ (Linde, 4.5 purity) and Ar (Linde, 6.0 purity) gases were used to saturate and deaerate the electrolyte solution, respectively.

### 2.2 Electrochemical Measurements

Electrochemical measurements were conducted in a typical one-compartment electrochemical cell configuration using an Autolab potentiostat (Multi Autolab/M204). A polycrystalline copper disk (Cu_Poly_–φ 5 mm) (Pine Research), graphite (MaTecK), and commercial leak-free Ag/Ag^+^ (Alvatek) were used as working, counter, and reference electrodes, respectively.

Prior to use, the Cu electrode was mechanically polished to a mirror finish using aqueous diamond pastes (Buehler, MetaDi 3, 1, 0.25, and 0.05 μm), rinsed with ultrapure water, and sonicated for 15 min to remove all residual mechanical polishing. Afterward, the electrode was electropolished in a 66% H_3_PO_4_ (Alfa Aesar, 85%) aqueous solution. Before each experiment, the copper surface was characterized by running a blank cyclic voltammogram (CV) in 0.1 M NaOH (Figure S1) at 50 mV/s, in a one-compartment cell configuration, using a Pt wire and a reversible hydrogen electrode (RHE) as counter and reference electrodes, respectively.

Before each experiment, the electrolyte solution was deaerated with Ar or saturated with CO_2_ for at least 20 min. During the measurements, the gas flow was kept above the electrolyte to avoid oxygen diffusing into the solution. Cyclic voltammograms were recorded at a flow rate of 50 mV/s.

All the potential values (*E*) were corrected for ohmic drop according to eq 1:
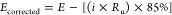
5where *R*_u_ is the uncompensated resistance measured.

### 2.3 Electrochemical CO_2_ Reduction Reaction

To investigate the products from the CO_2_RR, chronoamperometry experiments were carried out in a homemade two-compartment electrochemical cell configuration, wherein the outlet was directly connected to the gas chromatograph. The potential was controlled using an Ivium potentiostat (Vertex.50 V1A). A copper wire (MaTecK, 99.99% metal purity), which was also electropolished following the same procedure described before, used as the working electrode and the reference electrode (leak-free Ag/Ag^+^) were placed in the cathodic compartment, while the counter electrode, graphite (MaTecK), was placed in the anodic compartment. A proton exchange membrane (Nafion 117) was used to separate the cathodic and anodic compartments, which were filled with 8.0 mL of the electrolyte. Prior to the use of the membrane, it was rinsed with dry MeCN and soaked in dry MeCN for 1 h to remove the residual water. Before each experiment, CO_2_ was bubbled through the electrolyte for at least 15 min to reach the CO_2_ saturation. During the measurements, Ar and CO_2_, at a flow rate of 10 mL/min, were continuously bubbled through the anolyte and catholyte, respectively.

The CO_2_ gas flow rate in the inlet was controlled using Brooks Instruments. Any possible leakage in the cell was checked beforehand with the closed cell (without running electrolysis) by bubbling CO_2_ under controlled flow and analyzing the amount of the gas in the inlet and outlet.

The gaseous products from the electrolysis were analyzed using an online GC (Agilent, 990 Micro GC System), equipped with two thermal conductivity detectors (TCD), using Ar (channel 1) and He (channel 2) as carrier gases. A molecular sieve column combined with a Porabond Q precolumn (channel 1) was used to separate H_2_, O_2_, N_2_, CH_4_, and CO, while a CP-PoraPLOT U column (channel 2) was used to separate CO_2_ and C_2_H_4_ on the other TCD. The peak intensity of each product was measured at 5 min intervals over a total duration of 120 min, as was the current, which is depicted in Figures S6 and S7. These measurements were taken at the time of detection and utilized to calculate the Faradaic efficiency according to eq 1, where *z* is the number of electrons, *n* is the number of moles measured in the GC, *F* is the Faraday constant, and *Q* is the total charge. The average Faradaic efficiency (FE%) was then plotted against various electrolyte conditions, which will be discussed subsequently.

Liquid products were analyzed using high-performance liquid chromatography (HPLC Agilent 1260, Infinity II) equipped with an Aminex HPX-87H ion exclusion column (Bio-Rad), a refractive index detector (RID, G7162A), and a variable wavelength detector (VWD, G7114A). The analyses were performed at 35 °C and 0.6 mL/min flow rate using 5 mM H_2_SO_4_ as eluent. A 50 μL sample was collected after 2 h and diluted with water to a final volume of 1.0 mL in a vial. The Faradaic efficiency of the liquid products was determined using a methodology analogous to that employed for the gas chromatography (GC) analysis.
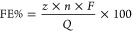


### 2.4 In Situ Fourier Transform Infrared Spectroscopy

In order to investigate the surface-adsorbed intermediates and products arising from the electrochemical CO_2_RR as a function of potential and different TAA^+^ cations, we conducted electrochemical in situ Fourier transform infrared (FTIR) spectroscopy. The measurements were carried out using a Nicolet iS50 FTIR Spectrometer outfitted with a VeeMax III accessory, measured using a liquid-nitrogen-cooled MCT detector. The in situ experiments were executed within a three-electrode spectro-electrochemical cell, with a CaF_2_ prism affixed to the cell’s base and the working electrode pressed against the prism to obtain a thin-layer configuration. FTIR spectra were acquired within the wavenumber range of 4000 to 1000 cm^–1^ and were recorded at potentials varying from −0.5 to −2.5 V vs RHE, while operating under either Ar or CO_2_ atmospheres. Spectral resolution was set to 4 cm^–1^ and is presented in reflectance mode, calculated via *A* = −log(*R*/*R*_0_), where *R* and *R*_0_ represent reflectance corresponding to the single-beam spectra acquired at the sample and reference potentials, respectively. Downward-pointing negative bands signify species present on or near the electrode surface at the reference potential that are subsequently consumed at the sample potential. Conversely, upward-pointing positive bands denote the formation of species at the sample potential. All spectro-electrochemical experiments were conducted at room temperature, employing the nonaqueous Ag/Ag^+^ reference electrode and platinum coils as reference and counter electrodes, respectively. It is important to mention here that due to the thin-layer configuration, the currents are very low, and under this condition, Pt dissolution does not happen in acetonitrile solution.^35^

## 3 Results and Discussion

### 3.1 Effect of Tetra-Alkyl Cations under Low Proton Availability in MeCN

**Figure 1:**
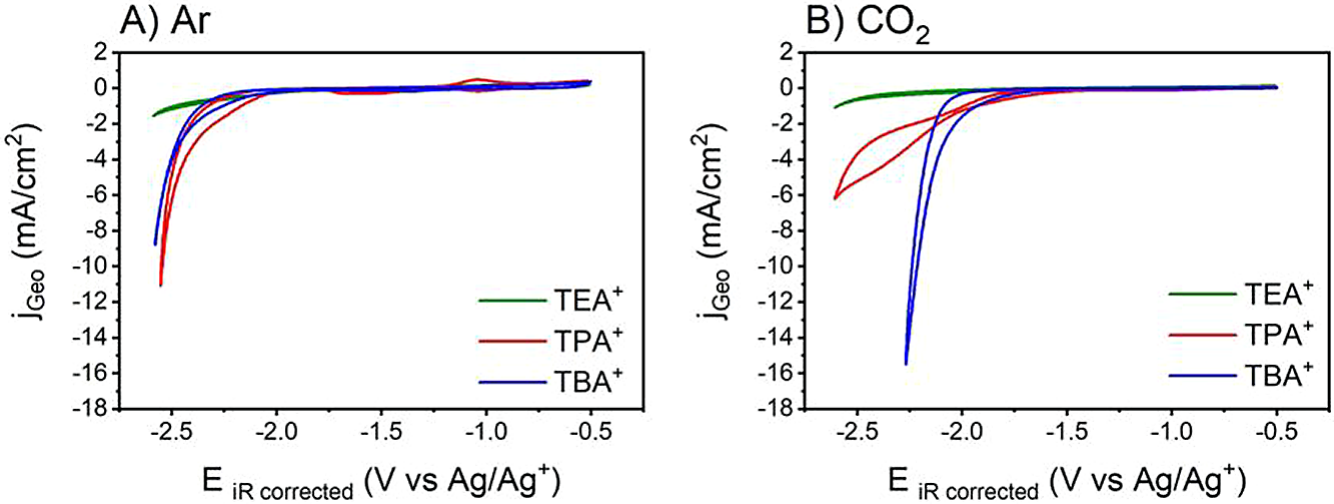
Cyclic voltammograms of the Cu_Poly_ electrode in 0.75 M TAAX in dry MeCN: (A) Ar-deaerated and (B) CO_2_-saturated solutions. Scan rate = 50 mV/s.

The role of the tetraalkylammonium cation (TAA^+^) in the CO_2_ reduction reaction (CO_2_RR) under low proton availability (dry) acetonitrile was investigated by cyclic voltammetry (CV) using polycrystalline Cu(Cu_Poly_). Figure 1 compares the cyclic voltammogram profiles in both Ar-deaerated (used as control) and CO_2_-saturated solutions, as shown in Figure 1A,B, respectively. It should be noted that, although strictly controlled for, water content in dry MeCN is relative given minor variations between experiments (Table S1).

We observe in both solutions, although more evident in Ar-deaerated solution, the presence of anodic and cathodic peaks with small current densities in the potential range between −0.5 and −1.2 V vs Ag/Ag^+^, which are attributed to the reduction of Cu(II) to Cu(I) and (Cu(I) to Cu(0), while the anodic peaks are attributed to the formation of a first layer of Cu(I) oxide and the formation of a second layer consisting of a mixture of Cu(II) oxide and hydroxide.^36^ The cathodic peak at potentials between −1.2 and −1.6 V is attributed to the hydrogen electro-adsorption, in this case from residual water, on the Cu electrode.^36−39^ We performed CV of a Cu electrode in Ar-deaerated solutions at different scan rates (Figure S2). We found that the peak currents of such processes are very small at a low scan rate indicating kinetic control of the processes; however, currents increase at a scan rate higher than 100 mV s^–1^, suggesting mass transport control.^40^ It means electroactive species are consumed more rapidly at the electrode surface.^40^

In Ar-deaerated solution (Figure 1A), no clear trend is observed between TPA^+^ and TBA^+^. The observed current density is likely due to the residual water present in the electrolyte (Table S1 and Figure S3) promoting HER.^13^ The degradation of acetonitrile also contributes to this effect.^41^ The higher currents observed for TPA^+^ and TBA^+^ in comparison to TEA^+^ (Figure 1A) are likely related to larger hydration shells of larger TAA^+^ cations due to the hydrophobic hydration,^42−45^ which facilitates HER.

More interestingly, in CO_2_-saturated solutions under low proton availability, the identity of the cation significantly affects the reduction current, which increases with the cation chain length. At electrode potentials more negative than approximately −1.5 V vs Ag/Ag^+^ (0.5 V less negative compared to the deaerated conditions), a notable increase in current density is observed, following the sequence TBA^+^ > TPA^+^ > TEA^+^. This observation substantiates the influence of the cationic chain length on the CO_2_RR activity in an acetonitrile solvent. Contrary, a comparison of the CVs at different cation concentrations during CO_2_RR does not show a significant change in the kinetics of the reaction (Figure S3), suggesting a common intermediate may be involved in the CO_2_RR reaction.

### 3.2 Effect of Tetraalkylammonium Cations under Higher Proton Availability in MeCN

**Figure 2:**
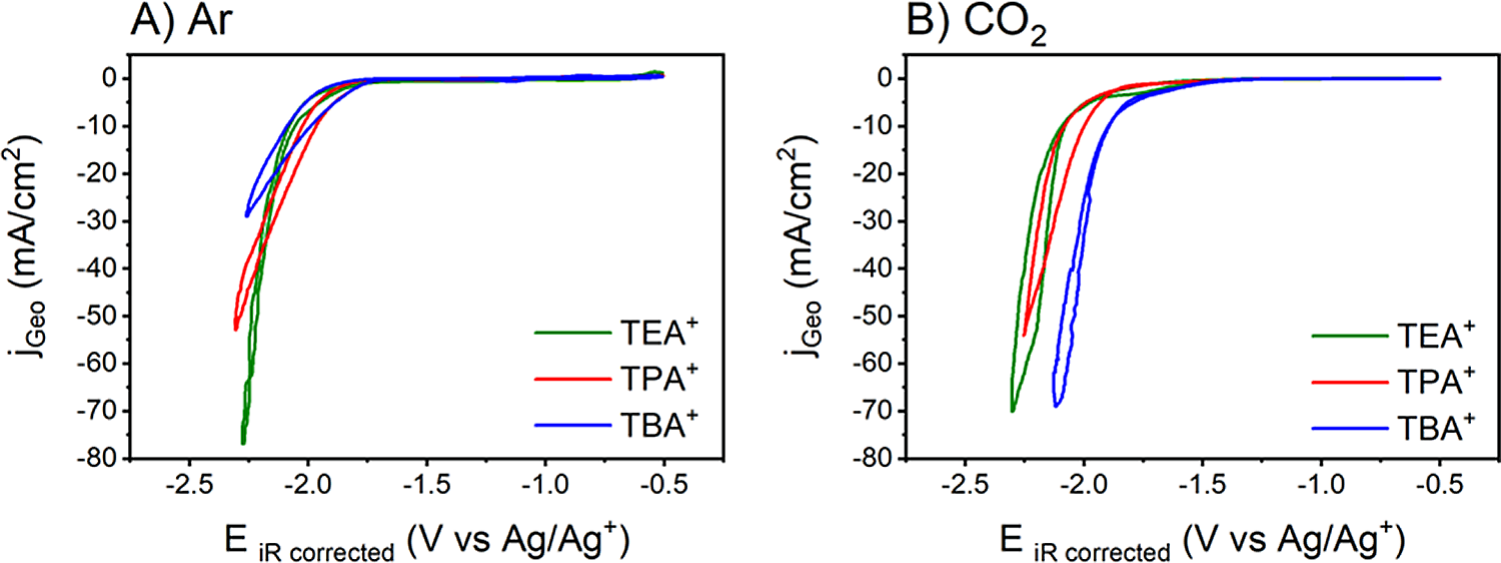
Cyclic voltammograms of the Cu_poly_ working electrode, with 0.75 M of different TAAX salts in wet MeCN: (A) deaerated with Ar; (B) CO_2_-saturated solutions. Scan rate = 50 mV/s.

From our previous study on nanostructured Cu electrodes, we found that the addition of water to the organic electrolyte influences the product distribution during CO_2_RR. Despite this, HER is inhibited even when 1000 mM H_2_O is added to the electrolyte solution.^13^ Given that the water content is important in determining the CO_2_RR selectivity, we also investigated the cation effect in acetonitrile electrolytes containing 1000 mM H_2_O (Figure 2). For simplicity, the results presented here will be described as “*wet*” MeCN solution.

Unlike under dry conditions, the cyclic voltammogram profiles do not show a clear trend in relation to the cation identity. However, higher current densities are observed in both Ar-deaerated and CO_2_-saturated solutions (Figure 2A,B, respectively) in comparison to the dry MeCN (Figure 1), likely due to the higher contribution of the competing water reduction reaction (2H_2_O + 2e^–^ → H_2_ + 2OH^–^).^36^

A comparison between dry and wet conditions when the electrolyte concentration is varied, Figures S3 and S4, respectively, shows that the ion cation concentration has no significant impact on the reactions under dry conditions, whereas there is a substantial effect under wet conditions (Figure S4). One possible explanation for such a result might be that the presence of water forms a hydration shell around the cations^32,42,45^ and that such a hydrated cation promotes the reaction better than cations itself.

We plotted the logarithm of the current density as a function of the logarithm of the concentration for each cation (Figure S5) under wet conditions for both Ar and CO_2_ solutions. We observe typically a positive slope, in both conditions, which suggest that hydrated cations enhance the rate of both HER and CO_2_RR, which could be, in this case, due to cations influencing the solvation and stabilization of intermediates at the electrode surface.^62^

### 3.3 Electrolysis Experiments

**Figure 3:**
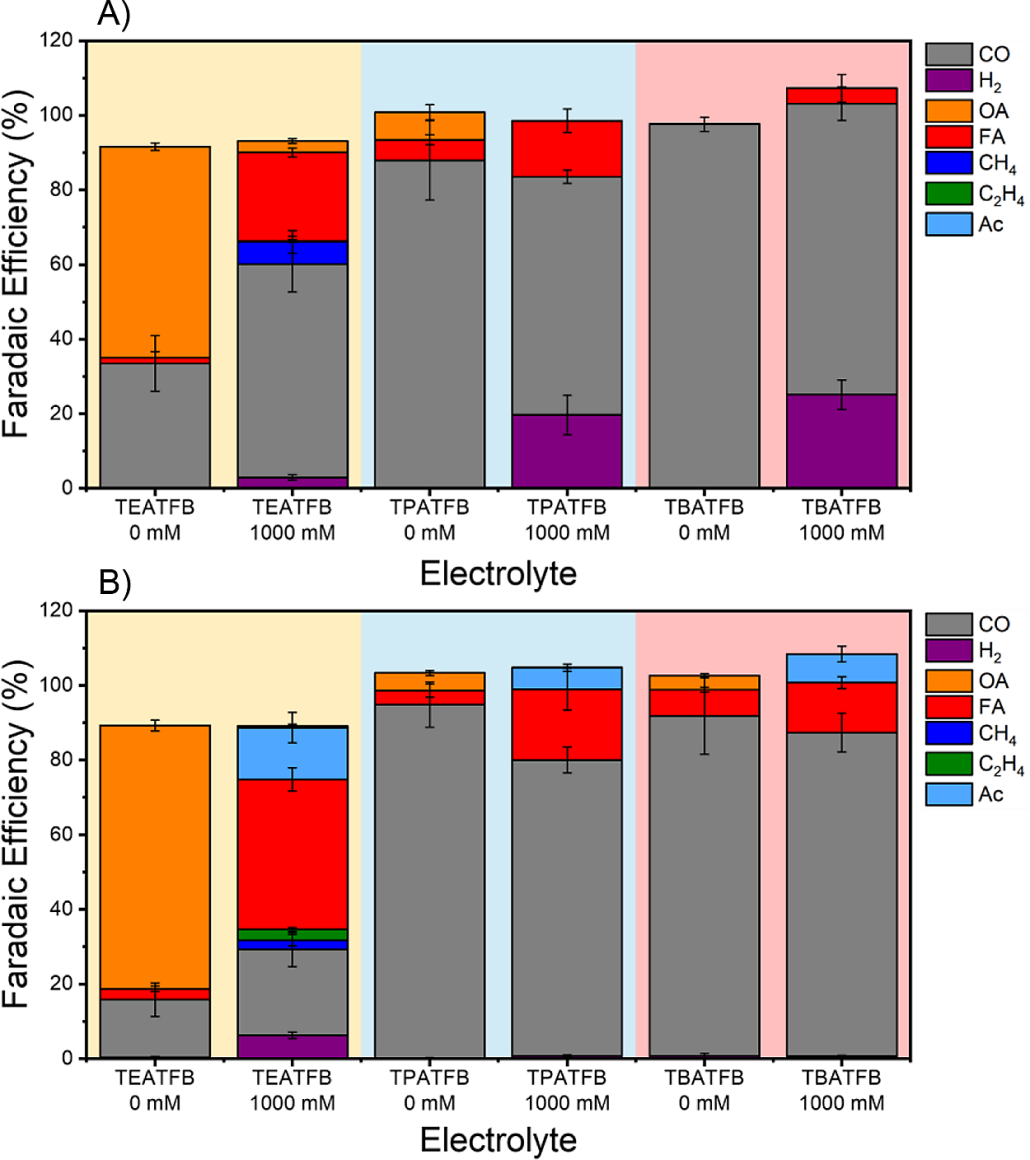
Faradaic efficiencies (FE%) of the Cu_poly_ electrode, 0.05 M TAAX (A) and 0.75 M TAAX (B) in both dry and wet MeCN. The yellow, blue, and red boxes display the FE% of TEATFB, TPATFB, and TBATFB, respectively. For each salt, dry conditions are displayed on the left, and wet conditions are displayed on the right. FE% was determined by online gas chromatography coupled with HPLC at −2.4 V vs Ag/Ag^+^ for dry conditions and at −2.1 V vs Ag/Ag^+^ for wet conditions. Chronoamperometry was performed for a total of 120 min. Products are defined as follows: OA - oxalic acid; Ac - acetic acid; FA - formic acid; C_2_H_4_ - ethylene; CH_4_ - methane; CO - carbon monoxide; and H_2_ - hydrogen.

To probe the effect of cation identity on the product distribution during the CO_2_RR in both, *dry* and *wet* MeCN electrolytes, chronoamperometry combined with online gas chromatography (GC) and high-performance liquid chromatography (HPLC) was carried out, at −2.4 V vs Ag/Ag^+^ and −2.1 V vs Ag/Ag^+^ for 120 min, respectively. The experiments were performed at low (0.05) and high (0.75 M) TAAX concentrations. The specific current densities for each condition are presented in Figures S6 and S7, whereas the product distribution and their corresponding Faradaic efficiencies (FE%) are shown in Figure 3. Time-resolved FE% for these experiments are also presented in Figures S8 and S9, as are the partial current densities in Figure S10.

In general, a small variation in the currents is observed, especially for TBA^+^ and TPA^+^, independent of the cation concentration and water content. Such a variation might be related to electrode instability, common during electrochemical measurements and solvent degradation.

**Scheme 1:**
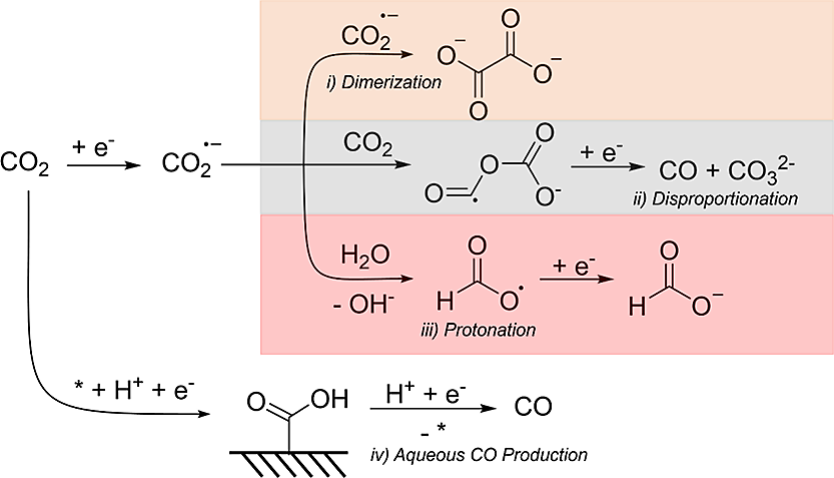
Mechanistic Pathways Available for CO_2_RR in Nonaqueous Aprotic Electrolytes The orange box describes oxalate formation via dimerization (step i), the gray box describes CO and CO_3_^2–^ formation via disproportionation (step ii), and the red box describes formate formation via protonation (step iii). The aqueous route for CO formation (step iv) is also included.

For TEA^+^, we observed an increase in current density as a function of time (Figures S6A,D and S7A). According to previous literature, an increase in current density would suggest an accumulation of active species at the interface, which means that under these conditions a higher local concentration of the CO_2_^·–^ intermediate. This intermediate is the key to promote the dimerization step (Scheme 1) which leads to the formation of oxalate, in agreement with our GC analysis as it will be further discussed.^19^

It is worthwhile to mention that the difference in current densities between CV (Figure 1B) and CA (Figure S7A) at the same potential is primarily due to the transient nature of CV and the steady-state conditions achieved in CA. The continuous potential sweep in CV leads to lower current densities, while the fixed potential in CA allows for higher steady-state currents due to more efficient mass transport and minimized capacitive effects.

Similar to previous literature on copper electrodes for the electrochemical reduction of carbon dioxide in the acetonitrile solvent,^13,18,20,46−48^ we found that carbon monoxide (CO) is a common product of the CO_2_RR, regardless of the cation identity or water content. We observe a significant increase in the Faradaic efficiency (FE%) of CO upon extending the hydrophobic tail from the TEA^+^ to the TPA^+^ cation. However, the further addition of a carbon atom in the tail from TPA^+^ to TBA^+^ results in a less pronounced increase in the CO FE%.

Waegele et al. previously demonstrated that in aqueous solutions, TAA^+^ cations do not block CO adsorption sites. However, larger cations like TPA^+^ and TBA^+^ more effectively displace interfacial water than smaller cations like TEA^+^. This displacement disrupts the hydrogen bonding between water and CO, which is crucial for stabilizing CO dimers, key intermediates in C–C coupling to ethylene.^49^ Smaller cations like TEA^+^, on the other hand, do not displace water as effectively, allowing the hydrogen bonding necessary to stabilize these dimers, thus promoting ethylene formation.

In contrast, our findings in acetonitrile solvent indicate a different behavior. Here, we observe that increasing the cation size from TEA^+^ to TBA^+^ does not follow the same trend as that in water. In acetonitrile, larger cations lead to less pronounced increases in the Faradaic efficiency (FE%) for CO formation beyond a certain point. This is attributed to the different solvation environment provided by acetonitrile, where the interfacial water content is significantly lower, and CO solubility is reduced compared to water. Under these conditions, and based on the findings previously obtained in aqueous solution,^49^ it seems that the Cu–CO interaction is weaker in acetonitrile solvent than in aqueous solvent,^49^ which likely contributes to the more facile CO desorption, and that the larger cations are more likely to disrupt the complex interfacial structure formed by the organic solvent and TAA^+^, leading to enhanced desorption of CO.

Scheme 1 illustrates the possible pathways available for the CO_2_RR in nonaqueous aprotic media. CO can be formed following two different steps; one in which water is not involved and CO_2_^·–^ intermediates follow a disproportionation reaction to carbon monoxide and carbonate species (step ii),^15^ and another one in which water is involved forming only CO (step iv).^24^ In agreement with the literature, when 1000 mM water is added to the electrolyte, we observe CO production. This is likely formed via a combination of steps ii and iv; however, the aqueous route competes with formic acid production via protonation reaction (step iii). This competition also extends toward acetaldehyde, ethylene, methane, and hydrogen, although these are minor products. HER is favored with larger cations, as expected for cations with larger hydration shells.^42^

Interestingly, the results in Figure 3 also show that small cations like (TEA^+^) promote better the CO_2_RR toward oxalate formation, where no water is involved (step i – Scheme 1), and CO_2_^·–^ radical combines via a dimerization reaction (step i).^19^ As the cation chain length increases, the FE% to oxalate significantly decreases, suggesting that under these conditions, the CO_2_ reduction reaction follows a step wherein water is involved in the mechanism of the reaction.

However, upon the addition of 1000 mM H_2_O to the supporting electrolyte, the FE% for OA decreases considerably, and therefore the protonation step (iii) to FA prevails over the dimerization reaction. The FE% to FA also increases following the order TEA^+^ > TPA^+^ > TBA^+^, suggesting that in the presence of water, the CO_2_RR mechanism favors the step involving protonation.

These results demonstrate that the influence of the TAA^+^ cation size on the CO_2_ reduction reaction mechanism significantly differs between aqueous and nonaqueous (acetonitrile) solvents, primarily due to the differing interactions between cations, solvent, water, and the reaction intermediates. For instance, in aqueous media, TBA^+^ cations form a 2D film at the electrode interface, affecting adsorbate coverage and thereby modifying catalytic activity. The formation of these films is influenced by the electrolyte’s composition and concentration, as well as the physical characteristics of the electrode surface.^50,51^

To support our results from Figure 3, we also compared the time-resolved dependency of chronoamperometry experiments with the FE% of the CO_2_RR products under both electrolyte conditions (Figures S8 and S9). Additionally, we plotted the partial current density of the products, which shows the same trend as that observed in Figure 3. Those results support our findings that CO production is favored on larger cations such as TPA^+^ and TBA^+^, whereas oxalate is the major product in the presence of the TEA^+^ cation.

Therefore, further investigations using in situ surface-enhanced infrared absorption spectroscopy (SEIRAS) or sum frequency generation (SFG) are necessary to elucidate cation adsorption dynamics.^33^

**Figure 4:**
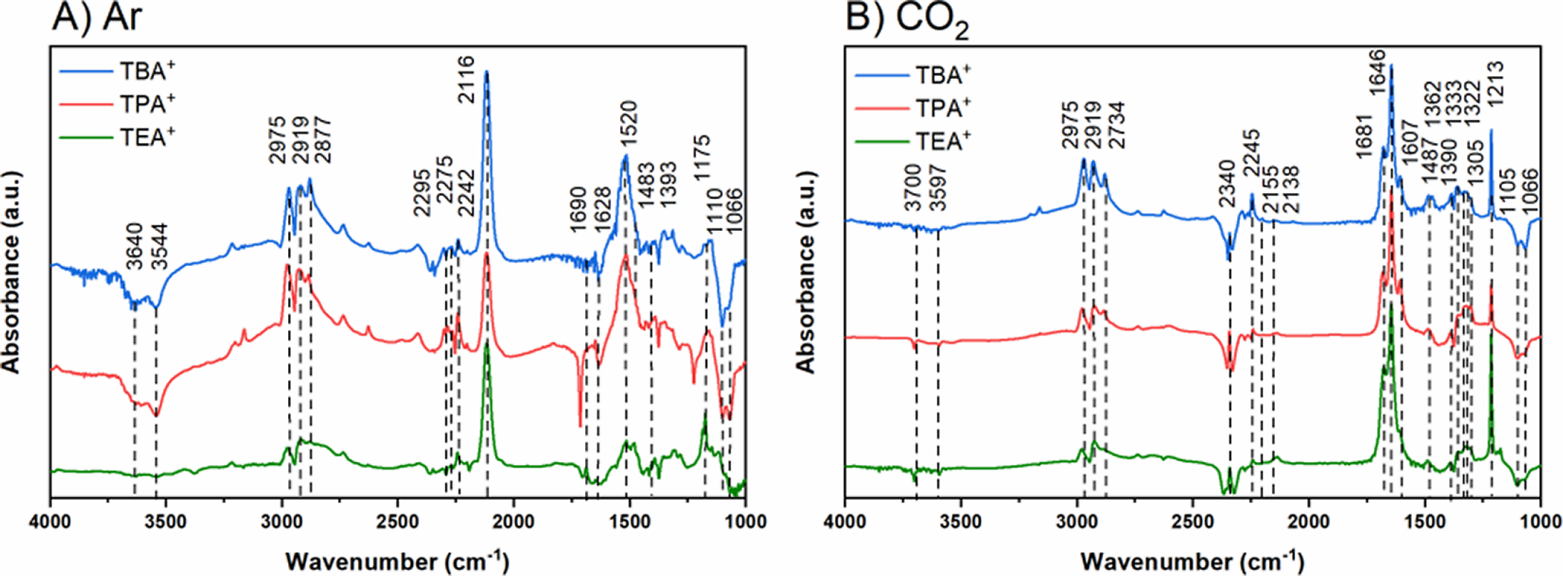
Electrochemical in situ FTIR spectra for dry 0.05 M TEATFB, TPATFB, and TBATFB MeCN electrolytes, in both (A) Ar-deaerated and (B) CO_2_-saturated conditions at applied potential of −2.4 V vs Ag/Ag^+^. Background spectra are recorded at −0.5 V vs Ag/Ag^+^.

**Scheme 2:**
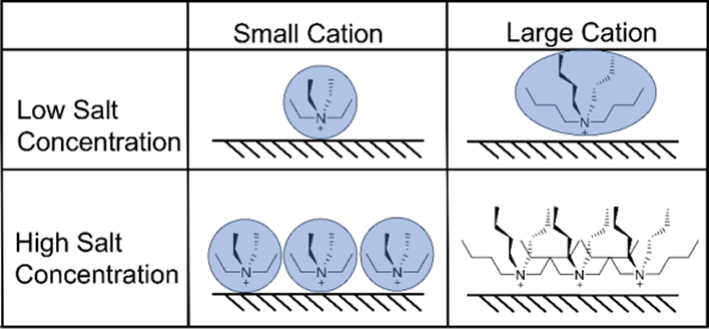
Tabular Representation of the Cation Interpenetration Effect Blue circles represent the hydration shell associated with the different cations on the cathode surface. Hydration shells are undisturbed until interpenetration occurs with large cations at high concentrations, which destroys the hydration shell.^32^

Regarding the experimental evidence of the presence of water and its interfacial state, in situ FTIR spectroscopy (Figure 4) supports the notion that in dry acetonitrile conditions, smaller cations like TEA^+^ result in reduced water content at the interface, therefore promoting oxalate formation. The spectra show significantly smaller bands related to water for TEA^+^ compared to those of larger cations, indicating less interfacial water. This supports the idea that limited water content close to the interface favors oxalate formation for smaller cations, aligning with our proposed mechanism in acetonitrile.

(19)Another interesting observation from the online GC data analysis is that at low cation concentration (0.05 M TAA^+^) (Figure 3A), hydrogen is only detected at a high concentration of water (1000 mM H_2_O); however, when the cation concentration increases to 0.75 M (Figure 3B), HER is completely absent for larger TPA^+^ and TBA^+^ cations, in agreement with the previous literature.^32^ Density functional theory (DFT) showed that high concentrations of cations with larger chain lengths will interpenetrate with one another at the interface and partially destroy the hydration shell. This creates an interfacial absence of water, restricting HER. This is why despite an increasing hydration shell, we observe less HER at high concentrations of salt. Therefore, it seems that our results suggest for such an interpenetration effect of adjacent cation alkyl chains.^32^ Unlike smaller space-filling cations such as TEA^+^, TBA^+^ is also penetrable. At high concentrations of a penetrable cation, steric interactions between aggregated cations occur given the strong electrostatic bias of the cathode.^32^ Given that the adsorbed cations are packed closely together on the cathode surface, longer alkyl chains are forced to be within close proximity. The alkyl chains of adjacent cations then penetrate each other. Considering that the hydration shell of tetraalkylammonium cations is distributed around the alkyl chains via hydrophobic hydration, the said hydration shell is disrupted and partially lost, preventing the HER at the cathode surface, as it will be further discussed by the in situ FTIR data. However, if smaller cations are present in high salt concentrations, then alkyl chains are not large enough to cause any significant disruption of adjacent cation hydration shells. Hence, this interpenetration effect is present in the case of both high concentrations and large chain length cations, as illustrated in Scheme 2.

The above consideration would explain why HER is largely suppressed as the cation chain length increases (TEA^+^ > TPA^+^ ≥ TBA^+^) when used at high concentrations, but HER is promoted when used in low concentrations (TEA^+^ < TPA^+^ < TBA^+^). As alkyl chain lengths increase, there is a corresponding rise in the number of water molecules that are associated with each cation.^42,45^ Thus, the availability of water in the Helmholtz plane increases, facilitating HER.^52^ Despite being the only other major product that is protonated, formate production is counter to this trend, with selectivity decreasing with chain length size. These two protonated species are in competition with one another, with formate being favored when local H_2_O concentrations at the interface are lower. This suggests that protonation of CO_2_^·–^ to produce formate occurs away from the cathode interface.

### 3.4 In Situ Fourier Transform Infrared Spectroscopy

To further investigate the relation between water in the proximity of the electrode surface and cation identity, we performed in situ FTIR spectroscopic analyses as a function of the applied potential.

A full set of spectra (Figures S11 and S12) and assignment tables (Tables S2 and S3) are available in the Supporting Information.

Figure 4 compares the spectra obtained at −2.4 V vs Ag/Ag^+^ for the Cu_poly_ electrode in the different electrolytes under Ar-deaerated and CO_2_-saturated dry solutions. The negative bands in the range of 3700 and 3500 cm^–1^, and 1620 cm^–1^ are assigned to the O–H stretching and bending modes of residual water being consumed,^53^ and they become more intense as the potential becomes more negative or as the concentration of water in the electrolyte increases (Figures S11 and S13). Although in situ FTIR was used as a qualitative identification of products and intermediates of the reaction, in Figure 4A, we observe that the intensity of those bands is significantly smaller for the smallest TEA^+^ cation, indicating reduced water content at the interface. This further supports the proposition that limited water content close to the interface is promoting oxalate formation for smaller cations.

Similar to our previous work, when CO_2_ is bubbled into the electrolyte solution (Figure 4B), the bands related to the water consumption are completely suppressed,^13^ independent of the cation identity, suggesting CO_2_RR inhibits HER. No hydrophobicity effect was considered as we would expect to see such an effect in Ar-deaerated solution as well and not only in the presence of CO_2_ solution. We also observe that the consumption of the residual water is accompanied by a concurrent gain of the positive bands at 2295, 2275, and 2245 cm^–1^, which are assigned to the C–N stretching of MeCN close to the electrode surface.^24^

Another common feature is the consumption of the bands at 1105 and 1066 cm^–1^, and the appearance of positive bands at 2975 and 2919 cm^–1^, (and 2877 cm^–1^) which are assigned to the B–F scissoring vibrations caused by the BF_4_^–^ anion and C–H stretching vibration caused by alkyl chains of TAA^+^ cations, respectively.^24^ At more positive potentials, the bands assigned to B–F are not visible (Figures S11 and S12) because of the background of the measurement which was also collected at more positive potential (−0.5 V vs Ag/Ag^+^). However, they become more visible and intense with increasingly negative potentials due to an increasing thickness of the diffusion layer, which is highly distorted in this configuration, caused by the change in the potential, meaning that within the thin-layer configuration, a larger proportion of cations are found at the electrode surface at the expense of anion.^24^ This suggests that the electrolyte structure changes near the electrode surface in response to the applied voltage, facilitating the migration of ion pairs rather than individual ions.^54^ In addition, the band at 2877 cm^–1^ is present only in TPA^+^ and TBA^+^, not in TEA^+^. This is due to the additional chemical environment caused by the larger chain length TAA^+^ cations.^55^

Despite such similarities, there are also critical differences between spectra shown in Figure 4A,B. In Ar-deaerated electrolytes, we only observe negative and positive bands at 1690, 1520, 1483, and 1175 cm^–1^, which are related to acetonitrile decomposition (negative band) and formation of acetamide (positive band), as has been previously reported.^41^ We also observe a strong band at 2116 cm^–1^ which is due to the C≡N stretch of the 3-aminocrotononitrile anion, formed by a dimerization between two acetonitrile molecules at exceedingly dry conditions (very low water ppm).^41^ The prior mentioned band at 1520 cm^–1^ is also caused by the conjugated C=N stretch of the 3-aminocrotononitrile anion.^41^ The presence of this species confirms that our electrolyte solution contains very low traces of water.

The same bands do not however appear when the electrolyte is saturated with CO_2_ (Figure 4B), indicating that CO_2_RR takes precedence over MeCN decomposition. Instead, positive bands are observed in the wavenumber range between 1700 and 1200 cm^–1^ and are assigned as carbonyl groups from CO_2_RR products.^24^ These bands increase in intensity as the band assigned to C=O stretching mode from CO_2_ at 2340 cm^–1^ becomes more negative as the potential increases (Figure S5).^24^ We observe all bands previously reported by Figueiredo et al.^24^ (Tables S2 and S5); however, the band at 1213 cm^–1^ is much larger in intensity relative to the other carbonyl bands. In addition, we also find that it is most intense in TEA^+^, while TPA^+^ and TBA^+^ show weaker responses. We therefore postulate that this band is oxalate. It should also be noted that the band at 1305 cm^–1^ associated with both C–O stretch vibrations in oxalate and carbonate is much larger in intensity relative to adjacent carbonyl bands than is expected of spectra consisting solely of carbonate.^24^ Therefore, this feature is likely formed by a combination of the aforementioned carbonyl bands appearing at the same frequency.^24,56^

**Figure 5:**
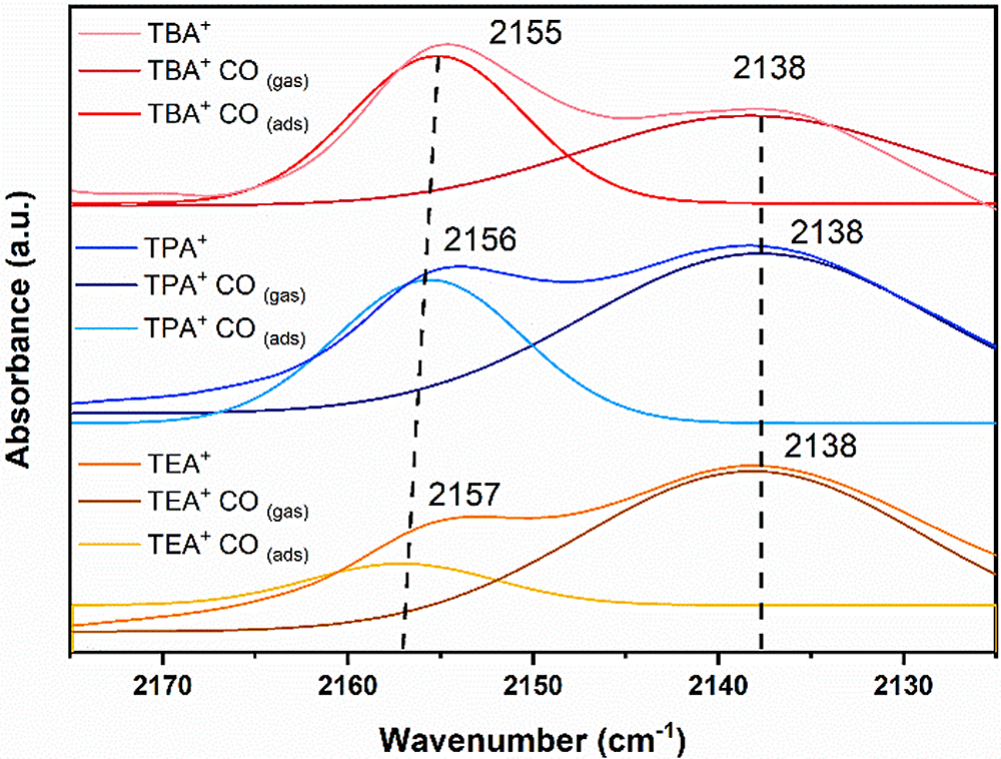
Electrochemical in situ FTIR spectra for dry 0.05 M TEATFB (orange line), TPATFB (blue line), and TBATFB (red line) MeCN electrolytes, in CO_2_-saturated conditions in the CO wavenumber region with applied potentials of −2.4 V vs Ag/Ag^+^. Background spectra are recorded at −0.5 V vs Ag/Ag^+^.

In addition to the aforementioned carbonyl bands, we could identify a band which is related to CO. In the CO_2_-saturated electrolyte, we observe two bands at 2155 and 2138 cm^–1^ at potentials more negative than −2.0 V vs Ag/Ag^+^ (Figure 5).

The broad band at 2138 cm^–1^ concerns to CO in the gas phase,^24^ which is an expected product from CO_2_RR as it is formed in equimolar amounts with the formation of CO_3_^2–^ via disproportion in the Amatore–Savéant mechanism,^15,19^ and such a band has previously been reported.^15,19,24^

Adjacent to CO_(gas)_, another band is detected at 2155 cm^–1^, which is attributed to the CO adsorption on the Cu surface.^57−60^ The observed redshift to marginally lower wavenumbers agrees with predictions based on the Stark effect,^57^ which finds that the resonant frequencies of oscillating dipoles are altered under the influence of an external electric field.

The spectra shown in Figure 5 indicate a (slightly) higher intensity for the band at 2155 cm^–1^ in the presence of larger cations such as TBA^+^ and TPA^+^ compared to TEA^+^, suggesting increased CO coverage at the electrode surface (disproportionation step illustrated in Scheme S1). Despite the inherently weak Cu–CO interactions in acetonitrile, which facilitate easier CO desorption,^13^ this observation correlates with the increased Faradaic efficiency (FE%) of CO for larger cations, as depicted in Figure 3. Nonetheless, the underlying mechanisms promoting enhanced CO adsorption on Cu surfaces in the presence of TBA^+^ and TPA^+^ remain elusive. It appears that these larger cations more effectively stabilize the adsorbed CO intermediate during the disproportionation process, albeit through weak interactions, and therefore, a further desorption step takes place. Conversely, the inferior stabilization of this key intermediate by TEA^+^ promotes oxalate production in the diffusion-reaction layer,^15,19^ as evidenced by our chromatographic data.

**Figure 6:**
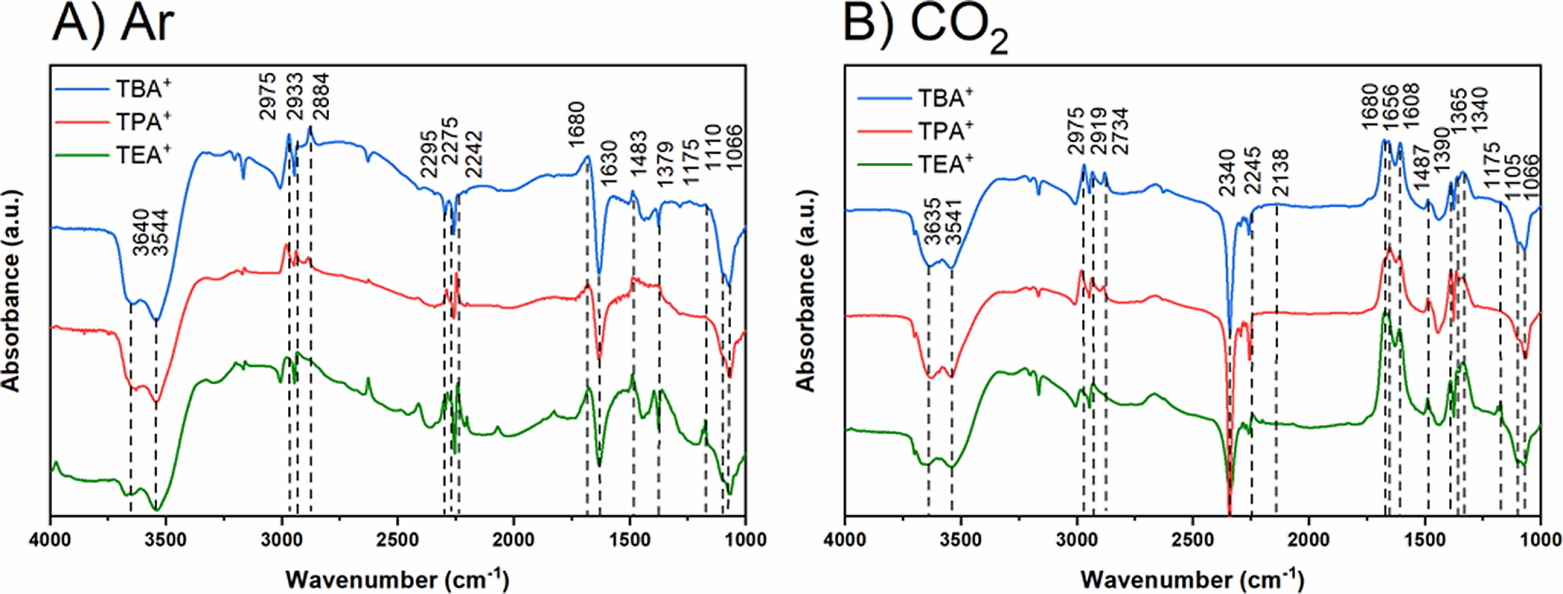
Electrochemical in situ FTIR spectra for wet 0.05 M TEATFB, TPATFB, and TBATFB MeCN electrolytes, in both (A) Ar-deaerated and (B) CO_2_-saturated conditions at applied potential of −2.4 V vs Ag/Ag^+^. Background spectra are recorded at −0.5 V vs Ag/Ag^+^.

Upon the addition of water to these electrolytes, the spectra shift, especially in the carbonyl region (Figure 6). A full set of spectra (Figures S13 and S14) and assignment tables (Tables S4 and S5) are available in the Supporting Information.

In Ar-deaerated conditions (Figure 6A), the bands at 2116 and 1520 cm^–1^ related to 3-aminocrotononitrile are no longer present. Instead, we observe much intensified bands at 3640 and 3544 cm^–1^, and 1630 cm^–1^ caused by the consumption of H_2_O. We also identify various positive bands, in particular 1680, 1485, and 1379 cm^–1^, which are related to the decomposition of MeCN to acetamide via the nucleophilic attack of electrogenerated OH^–^ on solvent molecules.^41^

Unlike in dry solvent, the spectra in CO_2_-saturated solvent under wet conditions (Figure 6B) show an intense negative band at 3640 and 3544 cm^–1^ related to the O–H stretching mode of H_2_O. The lack of significant differences in the water band region among various cations (TEA^+^ vs TBA^+^) might suggest a similar interaction of these cations with water molecules. However, this observation must be considered in the context of the experimental conditions, particularly the sensitivity and baseline stability of our in situ FTIR setup. The apparent similarity in the water band region can be influenced by instrumental limitations such as baseline drifting, which is a common issue in FTIR spectroscopy under varying environmental or experimental conditions, such as applied potential. As we already discussed, larger alkyl chain cations like TBA^+^ are expected to have a more pronounced hydrophobic effect, which disrupts the structured hydrogen bonding network of water more significantly than smaller cations like TEA^+^. Studies suggest that such disruption leads to a less structured, more loosely associated hydration shell around larger.^32,42−45^ This aligns with our hypothesis that TBA^+^ could facilitate more water molecules becoming available for the hydrogen evolution reaction (HER) under dry conditions, as a looser hydration shell may lead to increased water mobility at the electrode interface.

Spectra depicted in Figure 6 also show that a reduction in the number of carbonyl bands is observed. This is likely due to carbonate species being more readily protonate to bicarbonate. This causes an increase in signal intensity in the bands at 1608 and 1390 cm^–1^, as well as a merging of bands from 1365 to 1305 cm^–1^ which is caused by the reduction of different (bi)carbonate solvation modes as water is the preferred solvent.^24^ We also note that the band at 1213 cm^–1^ is no longer visible. This further suggests that the assignment to oxalate is correct as oxalate is not formed in the presence of water.^15,19,28,61^

## 4 Conclusions

We have demonstrated through a combination of electrochemical measurements with online GC and HPLC, and in situ FTIR the strong dependency of cation identity in aprotic media on the activity and selectivity of the CO_2_RR. Similar to previous literature, carbon monoxide is a common product from CO_2_RR in acetonitrile solvent; however, larger cations (TBA^+^ > TPA^+^ > TEA^+^) favor its formation via steps ii and iv (Scheme 1), in dry and wet acetonitrile.

We find that the dimerization step to oxalate formation is promoted with smaller cations, while its formation is limited with larger cations TBA^+^. Oxalate production is also limited when electrolytes are wet, with protonation step to formate being favored instead. CO_2_RR selectivity is not strongly influenced by supporting electrolyte concentration but does completely arrest HER when larger cations are employed (TPA^+^ and TPA^+^). These results are consistent within the context of the hydrophobic hydration of tetraalkylammonium cations and play a critical role in determining selectivities. The subsequent local concentrations of H_2_O at the interface largely dictate the CO_2_RR pathway; however, further investigation involving in situ SFG analysis should be performed to better understand the adsorption of residual water and cations on the surface of the electrode. Furthermore, we observe bands related to adsorbed CO in the in situ FTIR spectra, providing further insights into how cations are involved in stabilizing such intermediates.

## Supporting Information Available

Additional cyclic voltammetry and reaction order figures, water content information, mechanistic schemes, chronoamperometries, partial current densities, time-resolved FE% plots, and in situ FTIR spectra with assignment tables (PDF)
