# Supplementary material for: The Effect of the Tetraalkylammonium Cation in the Electrochemical CO2 Reduction Reaction on Copper Electrode
Source: ACS Catal. 2024 Aug 14;14(17):12928–39. doi: 10.1021/acscatal.4c02297 (PMC11385355; doi:10.1021/acscatal.4c02297)
Supplement: Supplementary file 1 — cs4c02297_si_001.pdf [file cs4c02297_si_001.pdf]

The Effect of the Tetraalkylammonium Cation in the Electrochemical CO_2 Reduction Reaction on Copper Electrode

Connor Deacon-Price[1], Louis Changeur[1], Cássia S. Santana[1,§], Amanda C. Garcia[1,*]

Van't Hoff Institute for Molecular Sciences, University of Amsterdam, Science Park 904, 1098 XH, Amsterdam, The Netherlands

(*) Corresponding author: a.c.garcia@uva.nl

(§) Current address: HTC 31, Holst Centre / TNO &IMEC-nl, High Tech Campus 31, 5656AE, The Netherlands.

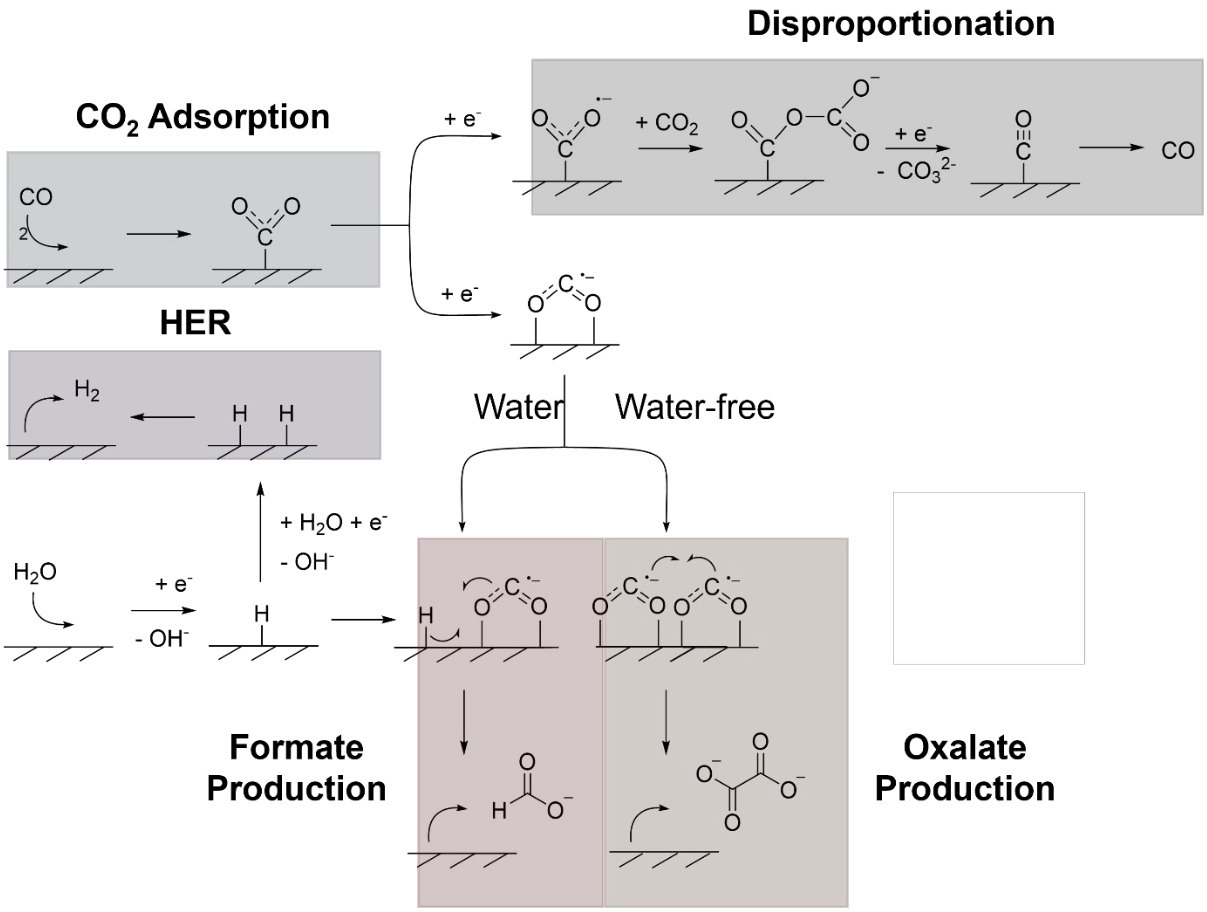

Scheme S1. A proposed mechanism for the formation of CO and CO_3^{2-} through disproportionation (grey box), formate through protonation (red box), oxalate through radical recombination and dimerization (orange box), and hydrogen through HER (purple box) within a CO_2 adsorption-based regime.

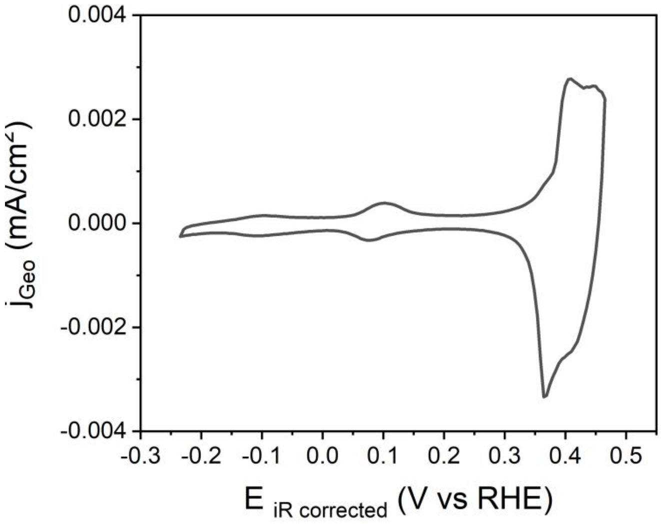

Figure S1. Cyclic voltammogram (CV) of polycrystalline Cu (Cu_{Poly}) in 0.1 M NaOH, under an Ar atmosphere, at a scan rate of 50 mV/s. CE = Pt_{Poly}; RE = RHE.

Table S1 Table representing the water content in ppm, recorded by Karl-Fischer Titration, of each measured electrolyte condition when using dry MeCN.

Salt	Concentration (M)	H_2O content in Ar (ppm)	H_2O content in CO_2 (ppm)
TEATFB	0.05	21.0	21.0
TEATFB	0.10	25.4	25.4
TEATFB	0.50	25.8	25.8
TEATFB	0.75	31.1	31.1
TPATFB	0.05	19.0	16.3
TPATFB	0.10	18.2	14.3
TPATFB	0.50	4.0	23.1
TPATFB	0.75	7.4	18.8
TBATFB	0.05	16.0	17.2
TBATFB	0.10	12.4	12.8
TBATFB	0.50	17.4	21.3
TBATFB	0.75	17.1	17.1

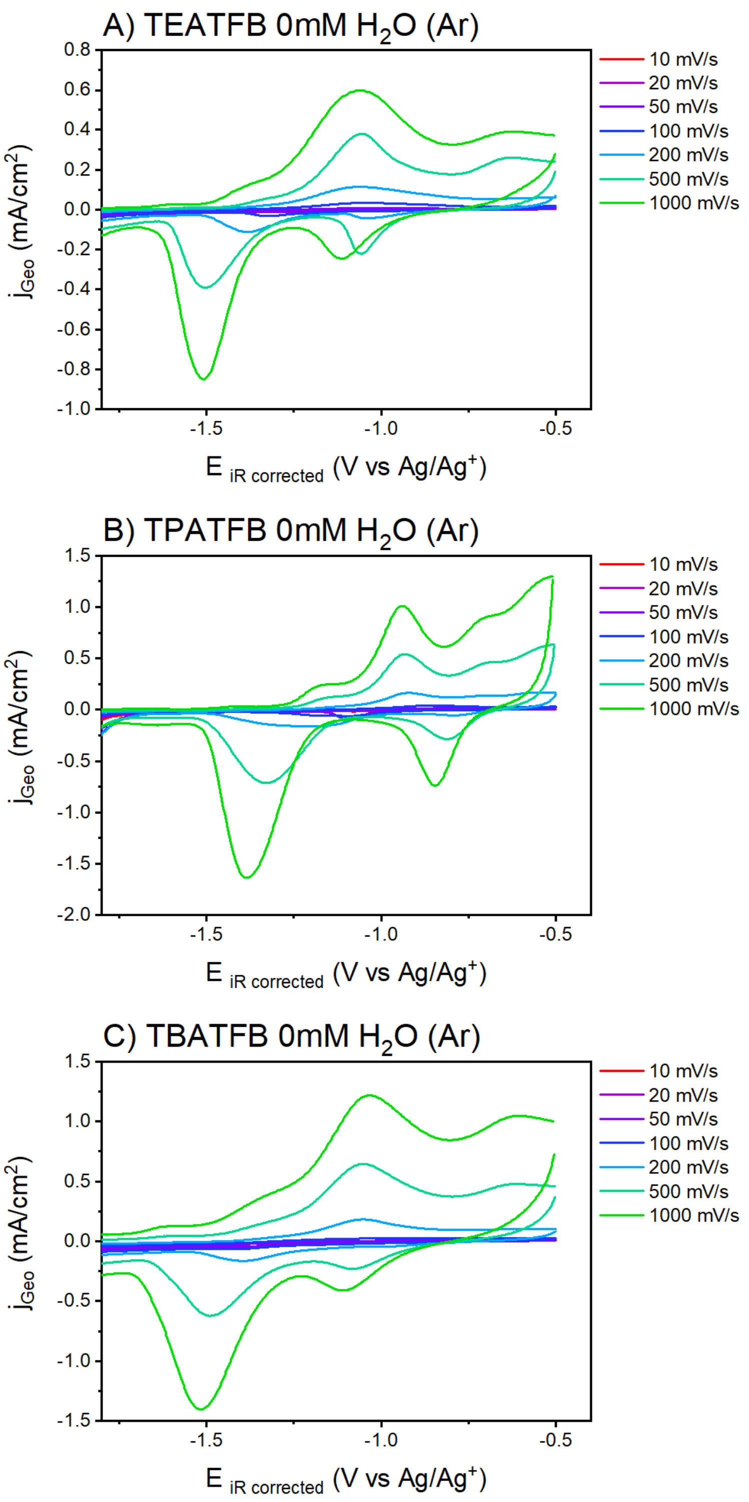

Figure S2. Cyclic Voltammograms of the CuPoly electrode, in A) 0.10 M TEATFB, B) 0.10 M TPATFB, and C) 0.10 M TBATFB in Ar deaerated conditions at various scan rates (10, 20, 50, 100, 200, 500, 1000 mV/s). CE = graphite, RE = commercial leak-free Ag/Ag$^+$

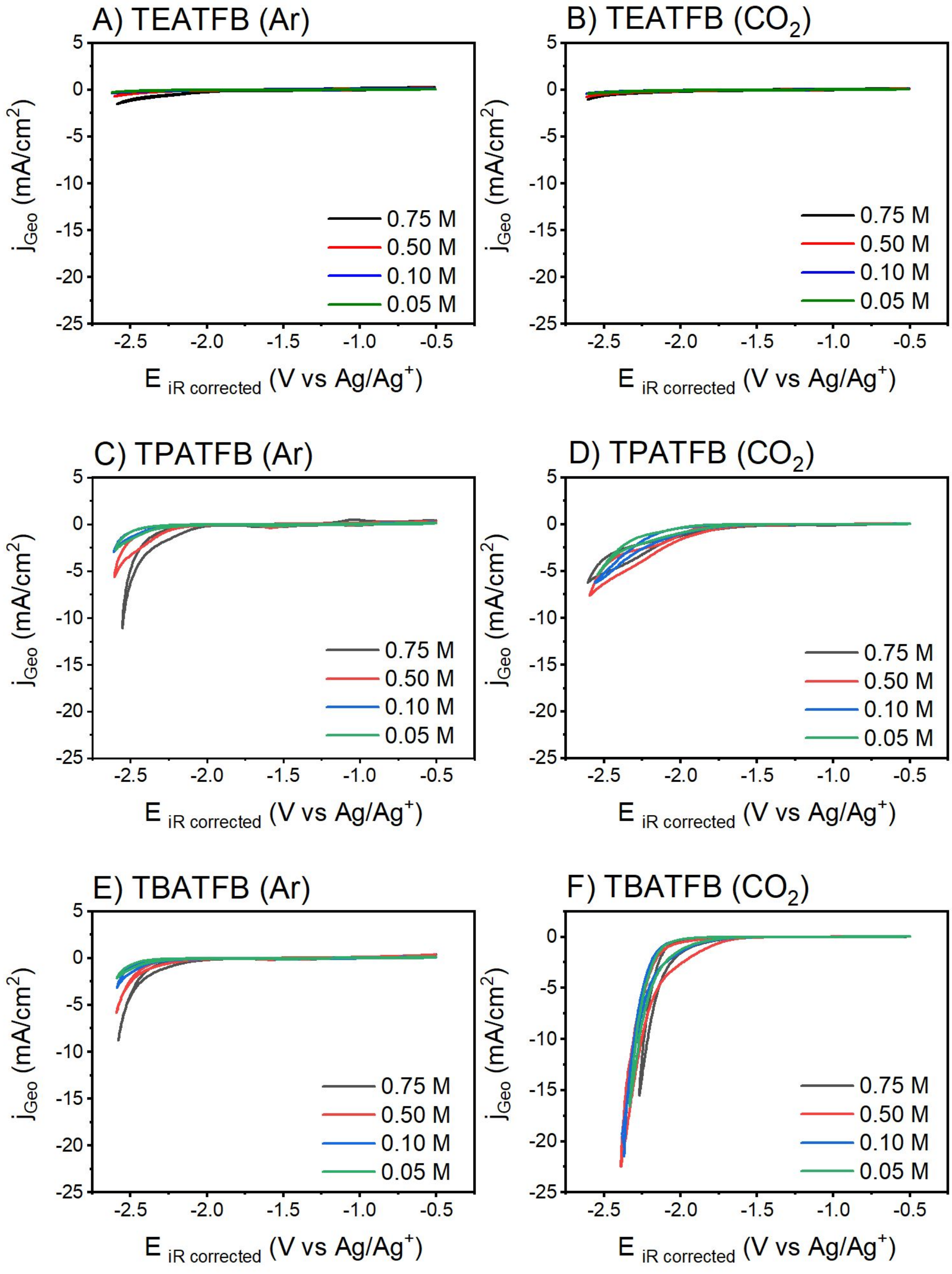

Figure S3. Cyclic Voltammetry (CVs) of Cu_{poly} electrode, varying concentrations of TAAX salts in dry MeCN: A) TEATFB solution deaerated with Ar; B) CO_2 saturated TEATFB solution; C) TPATFB solution deaerated with Ar; D) CO_2 saturated TPATFB solution; E) TBATFB solution deaerated with Ar; F) CO_2 saturated TBATFB solution, at a scan rate of 50 mV/s.

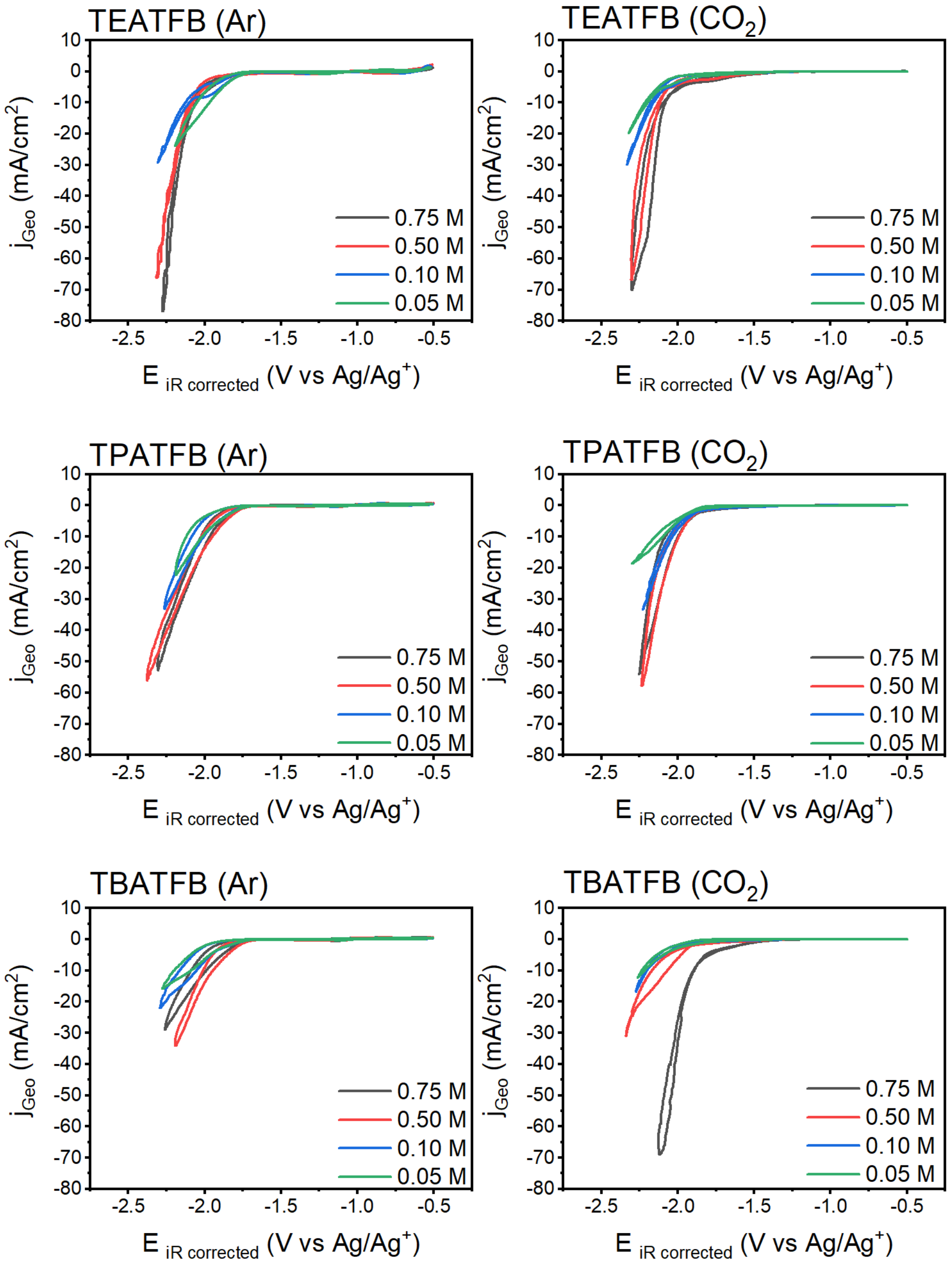

Figure S4. Cyclic Voltammetry (CVs) of Cu_{poly} electrode, varying concentrations of TAAX salts in wet MeCN (1000 mM H_2O addition): A) TEATFB solution deaerated with Ar; B) CO_2 saturated TEATFB solution; C) TPATFB solution deaerated with Ar; D) CO_2 saturated TPATFB solution; E) TBATFB solution deaerated with Ar; F) CO_2 saturated TBATFB solution, at a scan rate of 50 mV/s.

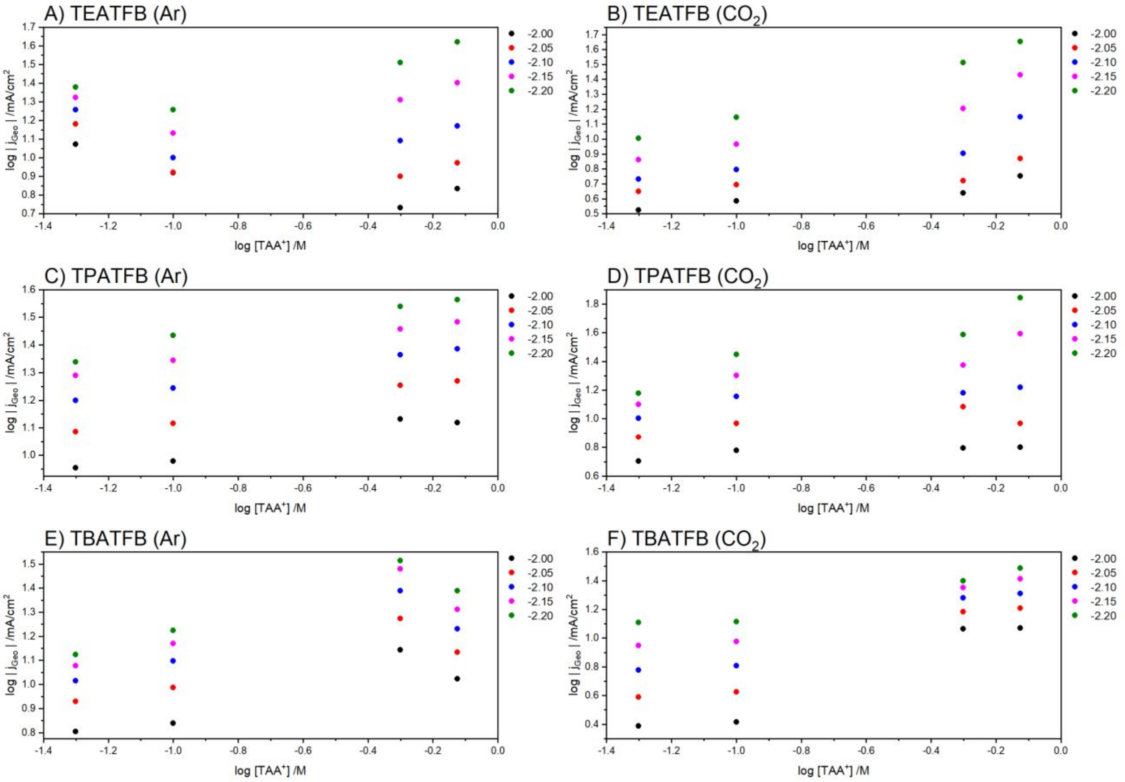

Figure S5. Reaction orders derived from cyclic voltammetry (CVs) of Cu_{poly} electrode, varying concentrations of TAAX salts in wet MeCN (1000 mM H_2O addition): A) TEATFB solution deaerated with Ar; B) CO_2 saturated TEATFB solution; C) TPATFB solution deaerated with Ar; D) CO_2 saturated TPATFB solution; E) TBATFB solution deaerated with Ar; F) CO_2 saturated TBATFB solution, at a scan rate of 50 mV/s.

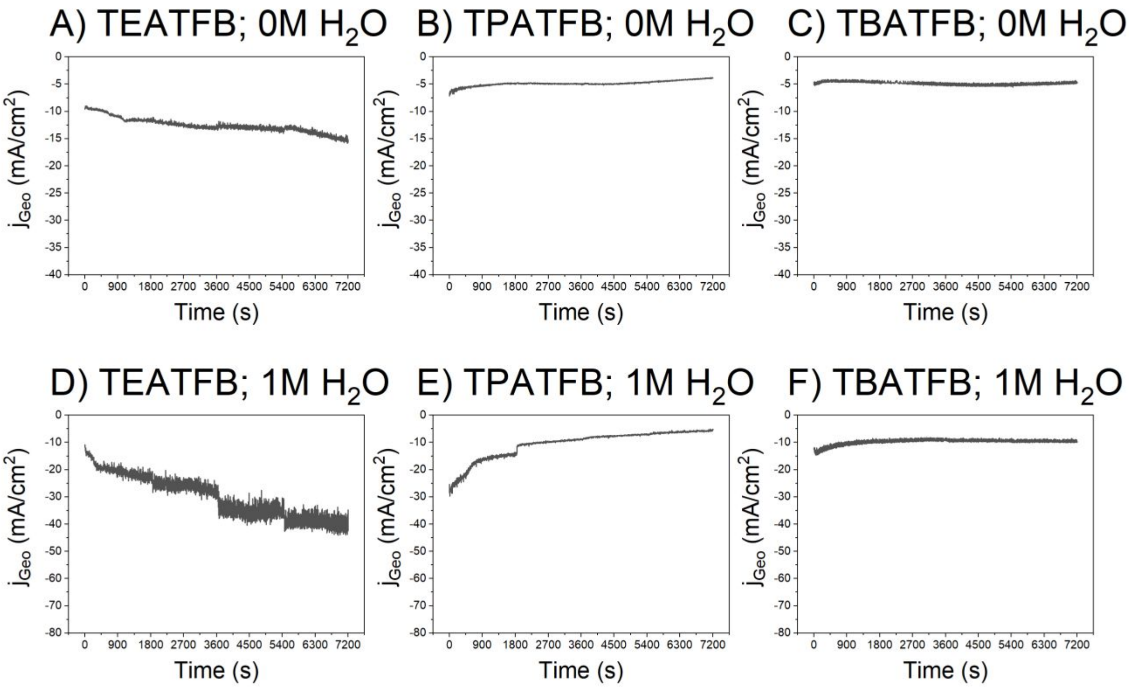

Figure S6. Chronoamperometry of Cu_{poly} electrode in dry CO_2 saturated MeCN solutions using supporting electrolytes A) 0.05 M TEATFB, B) 0.05 M TPATFB, and C) 0.05 M TBATFB and CO_2 saturated MeCN solutions with 1M water, using supporting electrolytes D) 0.05 M TEATFB, E) 0.05 M TPATFB, and F) 0.05 M TBATFB.

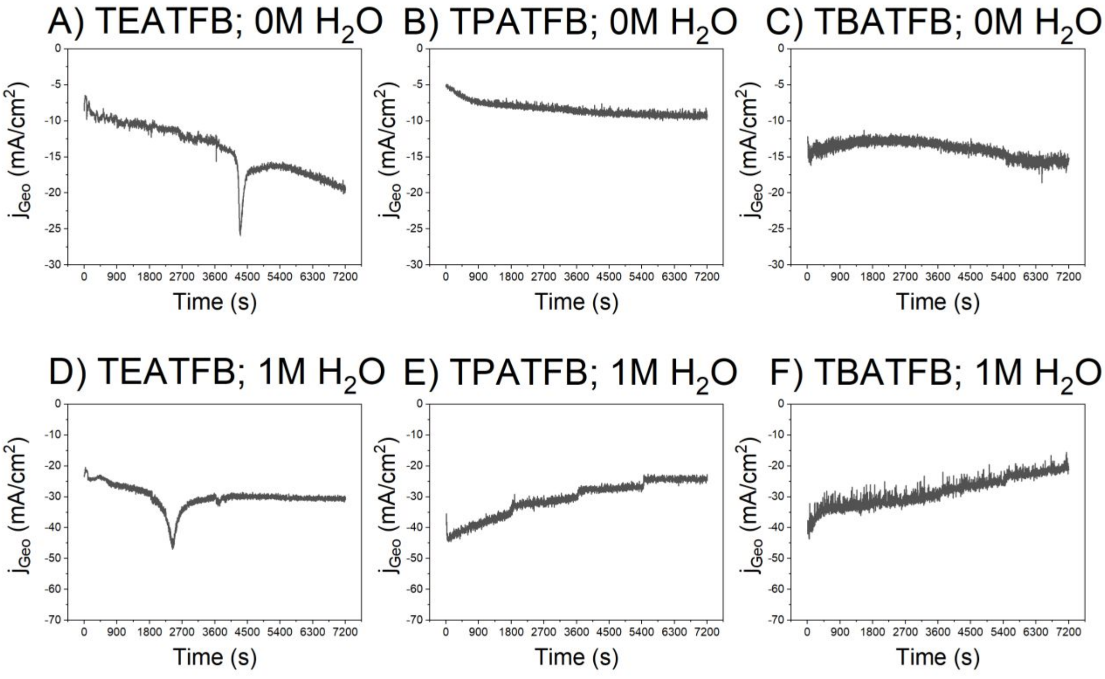

Figure S7. Chronoamperometry of Cu_{poly} electrode in dry CO_2 saturated MeCN solutions using supporting electrolytes A) 0.75 M TEATFB, B) 0.75 M TPATFB, and C) 0.75 M TBATFB and CO_2 saturated MeCN solutions with 1M water, using supporting electrolytes D) 0.75 M TEATFB, E) 0.75 M TPATFB, and F) 0.75 M TBATFB.

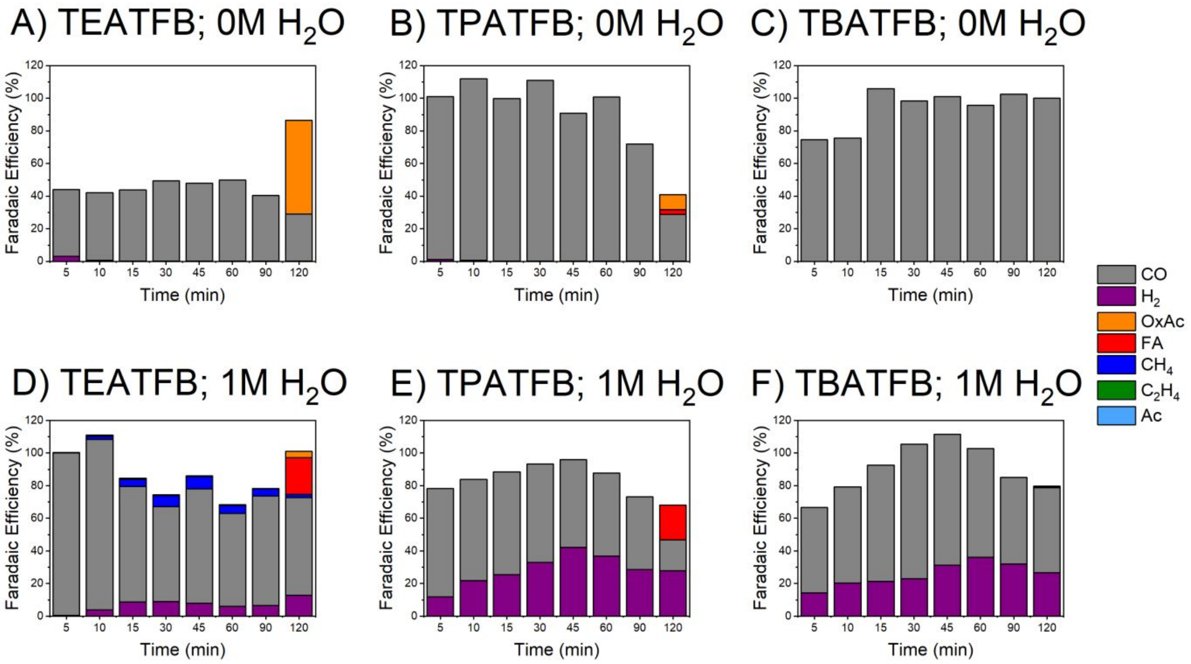

Figure S8. Time resolved Faradaic efficiencies (FE%) of Cu_{poly} electrode in dry CO_2 saturated MeCN solutions using supporting electrolytes A) 0.05 M TEATFB, B) 0.05 M TPATFB, and C) 0.05 M TBATFB and CO_2 saturated MeCN solutions with 1M water, using supporting electrolytes D) 0.05 M TEATFB, E) 0.05 M TPATFB, and F) 0.05 M TBATFB. FE% was determined by online gas chromatography coupled with HPLC at -2.4 V vs. Ag/Ag+ for dry conditions, and at -2.1 V vs. Ag/Ag+ for wet conditions. Chronoamperometry was performed for a total of 120 min. Products are defined as follows: OA - oxalic acid; Ac - acetic acid; FA - formic acid; C_2H_4 - ethylene; CH_4 - methane; CO - carbon monoxide; H_2 - hydrogen.

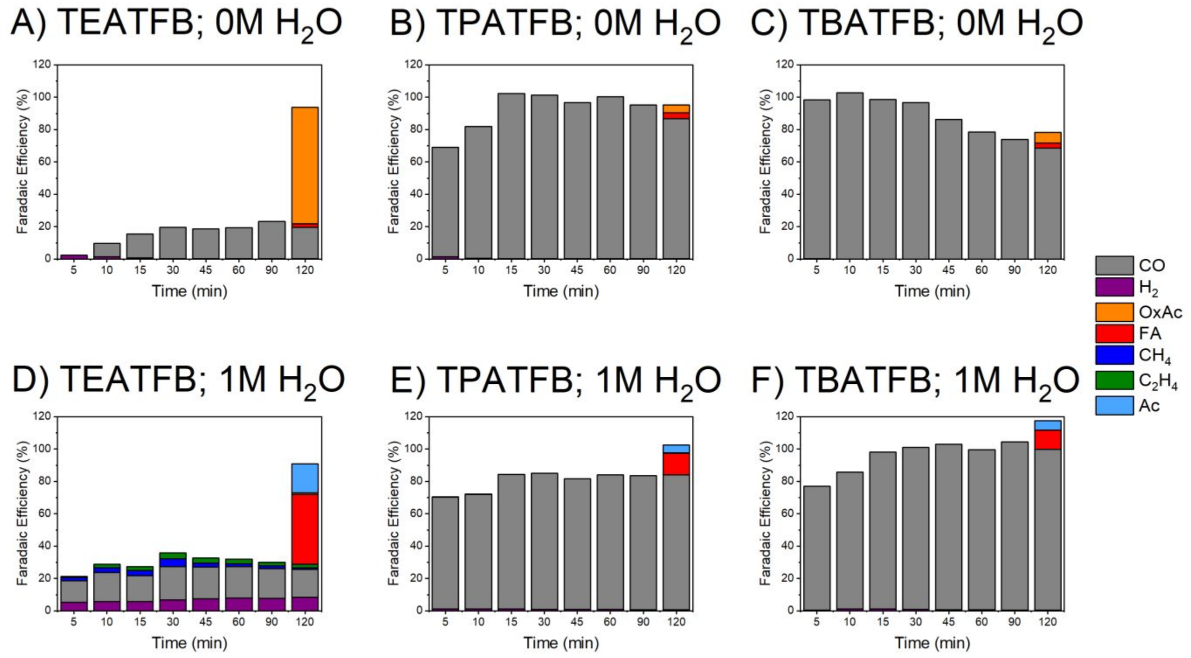

Figure S9. Time resolved Faradaic efficiencies (FE%) of Cu_{poly} electrode in dry CO_2 saturated MeCN solutions using supporting electrolytes A) 0.75 M TEATFB, B) 0.75 M TPATFB, and C) 0.75 M TBATFB and CO_2 saturated MeCN solutions with 1M water, using supporting electrolytes D) 0.75 M TEATFB, E) 0.75 M TPATFB, and F) 0.75 M TBATFB. FE% was determined by online gas chromatography coupled with HPLC at -2.4 V vs. Ag/Ag+ for dry conditions, and at -2.1 V vs. Ag/Ag+ for wet conditions. Chronoamperometry was performed for a total of 120 min. Products are defined as follows: OA - oxalic acid; Ac - acetic acid; FA - formic acid; C_2H_4 - ethylene; CH_4 - methane; CO - carbon monoxide; H_2 - hydrogen.

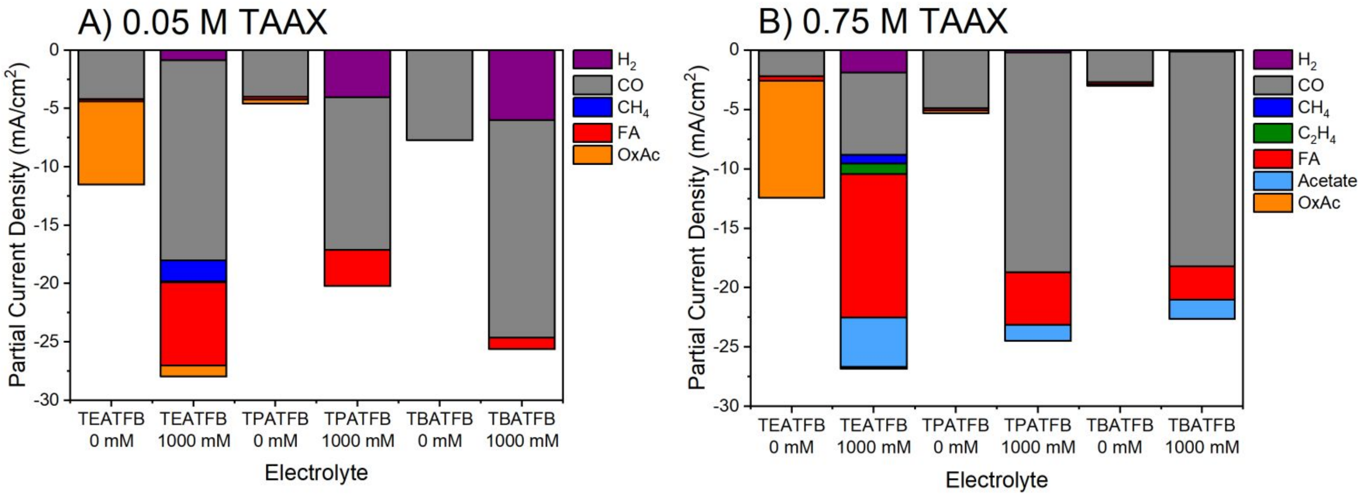

Figure S10. Partial current densities of Cu_{poly} electrode, 0.05 M TAAX (A) and 0.75 M TAAX (B) in both dry and wet MeCN. For each salt, dry conditions are displayed on the left, and wet conditions are displayed on the right. Chronoamperometry was performed for a total of 120 min. Products are defined as follows: OA - oxalic acid; Ac - acetic acid; FA - formic acid; C_2H_4 - ethylene; CH_4 - methane; CO - carbon monoxide; H_2 - hydrogen.

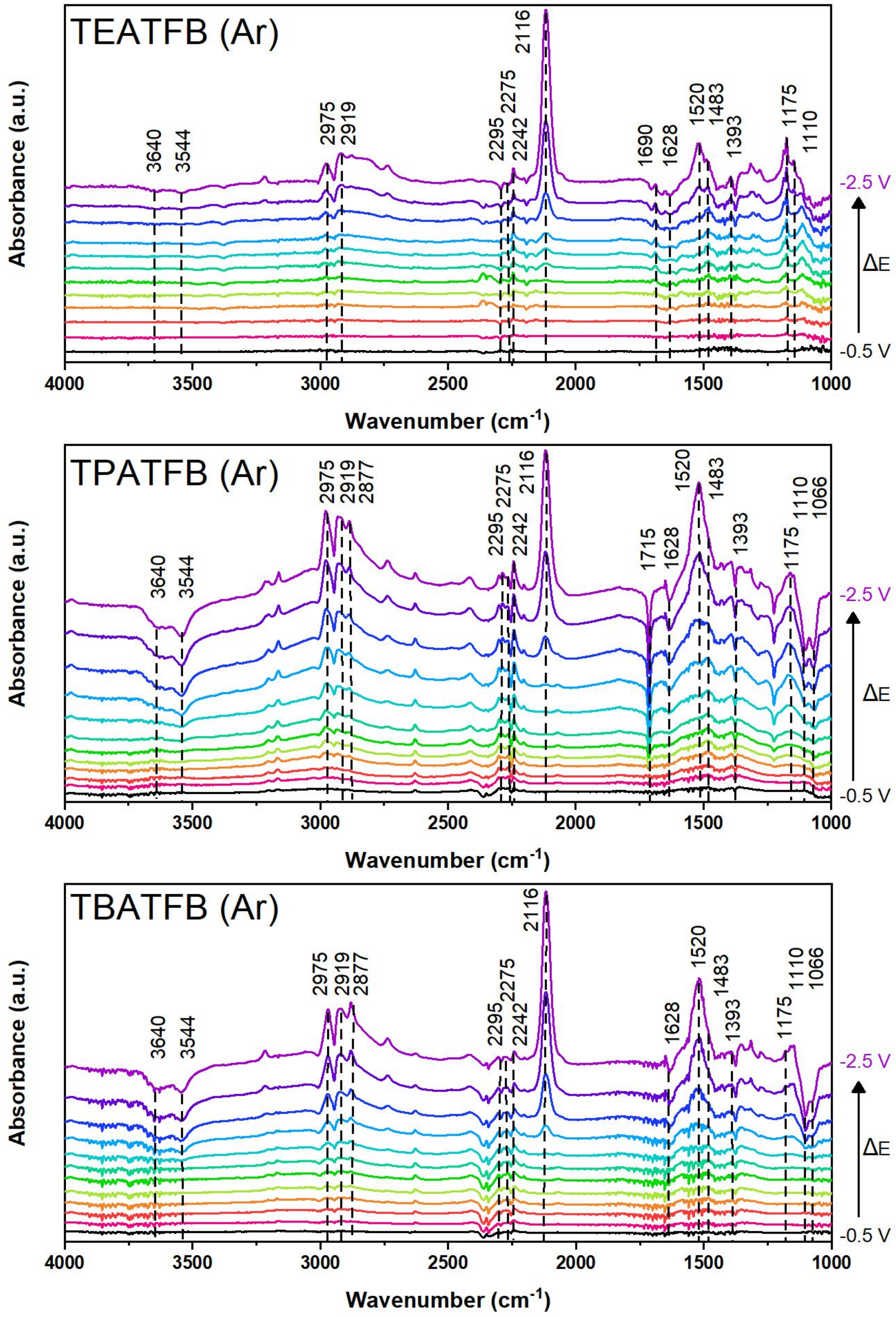

Figure S11. Electrochemical *in situ* FTIR spectra for dry 0.05 M TEATFB, TPATFB, and TBATFB MeCN electrolytes, in Ar deaerated conditions, at Cu_{Poly} WE. Background signals are recorded at -0.5 V vs Ag/Ag^{+}. Spectra are recorded at -0.5 V, then -1.0 V, increasing by -200 mV until -2.0 V, then increasing by -100 mV until -2.5 V.

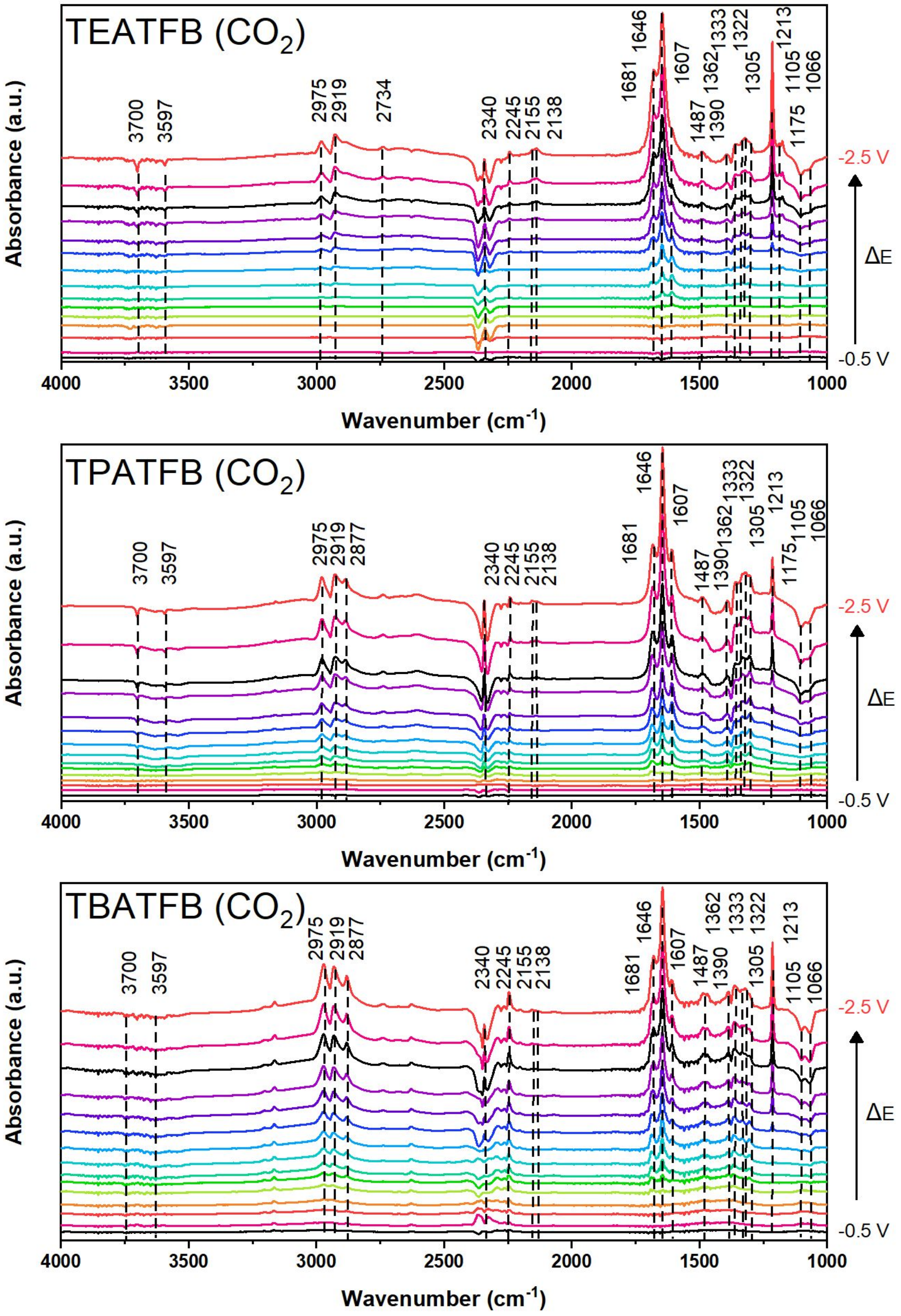

Figure S12. Electrochemical *in situ* FTIR spectra for dry 0.05 M TEATFB, TPATFB, and TBATFB MeCN electrolytes in CO_2 saturated conditions, at Cu_{Poly} WE. Background signals are recorded at -0.5 V vs Ag/Ag^+. Spectra are recorded at -0.5 V, then -1.0 V, increasing by -200 mV until -1.4 V, then increasing by -100 mV until -2.5 V.

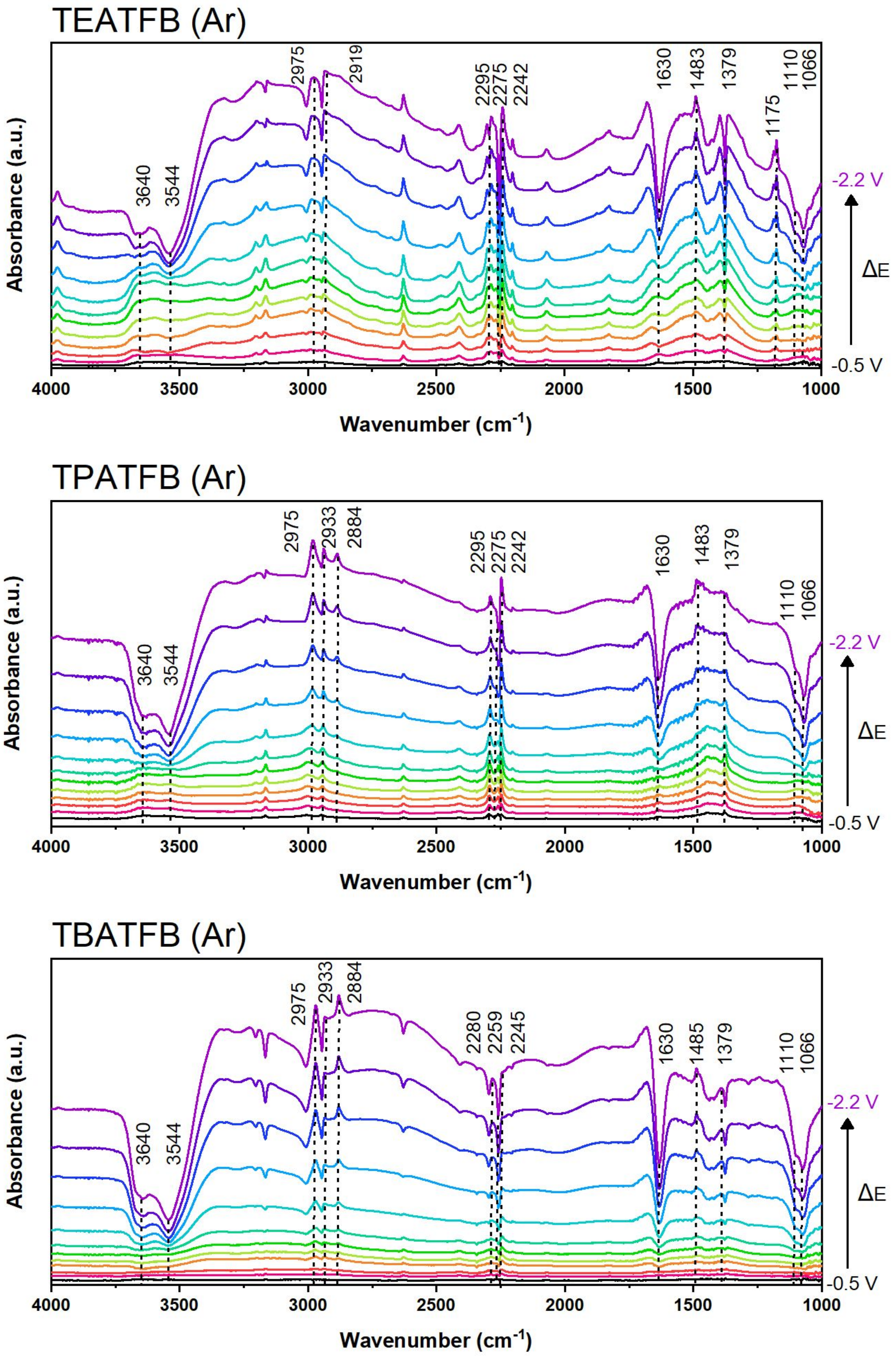

Figure S13. Electrochemical in-situ FTIR spectra for wet 0.05 M TEATFB, TPATFB, and TBATFB MeCN electrolytes, in Ar deaerated conditions, at Cu_{Poly} WE. Background signals are recorded at -0.5 V vs Ag/Ag^+. Spectra are recorded at -0.5 V, then -1.0 V, increasing by -200 mV until -1.4 V, then increasing by -100 mV until -2.2 V.

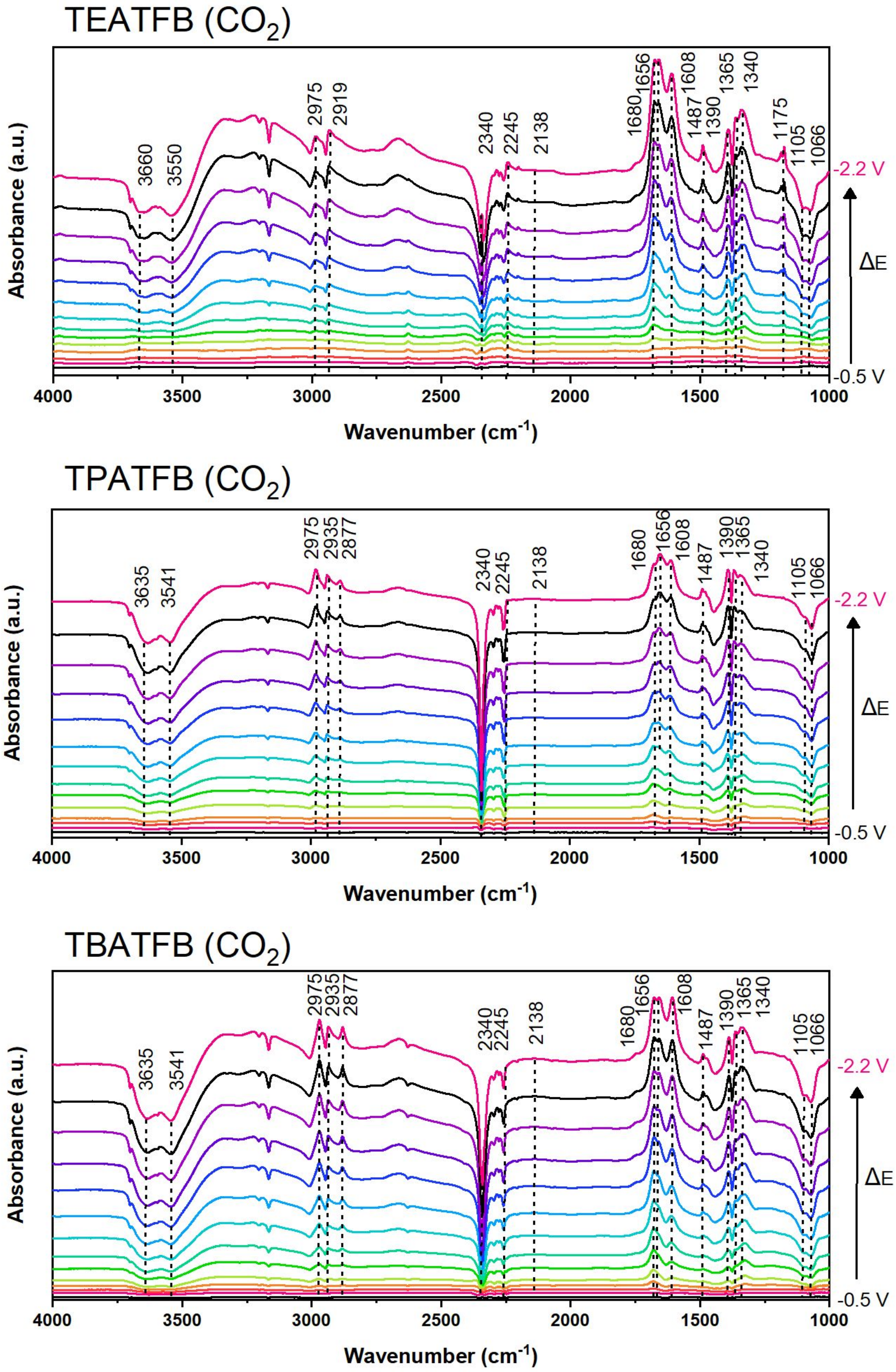

Figure S14. Electrochemical in-situ FTIR spectra for wet 0.05 M TEATFB, TPATFB, and TBATFB MeCN electrolytes, in CO_2 saturated conditions, at Cu_{Poly} WE. Background signals are recorded at -0.5 V vs Ag/Ag^{+}. Spectra are recorded at -0.5 V, then -1.0 V, increasing by -100 mV until -2.2 V.

Table S2. Assignment of the FTIR bands observed in dry and Ar deaerated MeCN electrolytes.

Wavenumber (cm^{-1})	Assignment	Compound	Reference
3640	ν O-H	H_2O	Figueiredo *et. al.*[1]
3544	ν O-H	H_2O	Figueiredo *et. al.*[1]
2975	ν C-H	TAA^+	Figueiredo *et. al.*[1]
2919	ν C-H	TAA^+	Figueiredo *et. al.*[1]
2877	ν C-H	TAA^+	Figueiredo *et. al.*[1]
2295	ν C-N	MeCN	Figueiredo *et. al.*[1]
2275	ν C-N	MeCN	Figueiredo *et. al.*[1]
2245	ν C-N	MeCN	Figueiredo *et. al.*[1]
2116	ν C≡N	3-Aminocrotononitrile	Foley *et. al.*[2]
1690	ν C=O	CH_3CONH_2	Foley *et. al.*[2]
1650	ν C=O	CH_3CONH_2	Foley *et. al.*[2]
1520	ν C=N	3-Aminocrotononitrile	Foley *et. al.*[2]
1483	δ CH_3	CH_3CONH_2	Foley *et. al.*[2]
1393	δ CH_3	CH_3CONH_2	Foley *et. al.*[2]
1175	δ NH_2	CH_3CONH_2	Foley *et. al.*[2]
1105	ν BF_4	BF_4^-	Figueiredo *et. al.*[1]

Table S3. Assignment of the FTIR bands observed in dry and CO_2 saturated MeCN electrolytes.

Wavenumber (cm^{-1})	Assignment	Compound	Reference
3700	ν O-H	H_2O	Figueiredo *et. al.*[1]
3597	ν O-H	H_2O	Figueiredo *et. al.*[1]
2975	ν C-H	TAA^+	Figueiredo *et. al.*[1]
2919	ν C-H	TAA^+	Figueiredo *et. al.*[1]
2877	ν C-H	TAA^+	Figueiredo *et. al.*[1]
2340	ν C=O	CO_2	Figueiredo *et. al.*[1]
2245	ν C-N	MeCN	Figueiredo *et. al.*[1]
2155	ν C-O	$Cu\text{-}{*}CO_{(ads)}$	This work, Moradzaman[3]
2138	ν C-O	$CO_{(gas)}$	Figueiredo *et. al.*[1]
1681	ν C=O	CO_3^{2-}	Figueiredo *et. al.*[1]
1646	ν C=O	CO_3^{2-}	Figueiredo *et. al.*[1]
1607	ν C=O	HCO_3^-	Figueiredo *et. al.*[1]
1487	δ CH_3	CH_3CONH_2	Foley *et. al.*[2]
1390	ν C-O	HCO_3^-	Figueiredo *et. al.*[1]
1362	ν C-O	CO_3^{2-}	Figueiredo *et. al.*[1]
1333	ν C=O	CO_3^{2-}	Figueiredo *et. al.*[1]
1322	ν C-O	CO_3^{2-}	Figueiredo *et. al.*[1]
1305	ν C-O	CO_3^{2-}	Figueiredo *et. al.*[1]
1213	ν C-H	HCO_3^-	Figueiredo *et. al.*[1]
1105	ν BF_4	BF_4^-	Figueiredo *et. al.*[1]
1066	ν BF_4	BF_4^-	Figueiredo *et. al.*[1]

Table S4. Assignment of the FTIR bands observed in wet and Ar deaerated MeCN electrolytes.

Wavenumber (cm^{-1})	Assignment	Compound	Reference
3640	v O-H	H_2O	Figueiredo *et. al.*[1]
3544	v O-H	H_2O	Figueiredo *et. al.*[1]
2975	v C-H	TAA^+	Figueiredo *et. al.*[1]
2933	v C-H	TAA^+	Figueiredo *et. al.*[1]
2884	v C-H	TAA^+	Figueiredo *et. al.*[1]
2295	v C-N	MeCN	Figueiredo *et. al.*[1]
2275	v C-N	MeCN	Figueiredo *et. al.*[1]
2245	v C-N	MeCN	Figueiredo *et. al.*[1]
1680	v C=O	CH_3CONH_2	Foley *et. al.*[2]
1630	δ O-H	H_2O	Foley *et. al.*[2]
1483	δ CH_3	CH_3CONH_2	Foley *et. al.*[2]
1379	δ CH_3	CH_3CONH_2	Foley *et. al.*[2]
1175	δ NH_2	CH_3CONH_2	Figueiredo *et. al.*[1]
1110	v BF_4	BF_4^-	Figueiredo *et. al.*[1]
1066	v BF_4	BF_4^-	Figueiredo *et. al.*[1]

Table S5. Assignment of the FTIR bands observed in wet and CO_2 saturated MeCN electrolytes.

Wavenumber (cm^{-1})	Assignment	Compound	Reference
3660	v O-H	H_2O	Figueiredo *et. al.*[1]
3550	v O-H	H_2O	Figueiredo *et. al.*[1]
2975	v C-H	TAA^+	Figueiredo *et. al.*[1]
2919	v C-H	TAA^+	Figueiredo *et. al.*[1]
2877	v C-H	TAA^+	Figueiredo *et. al.*[1]
2340	v C=O	CO_2	Figueiredo *et. al.*[1]
2245	v C-N	MeCN	Figueiredo *et. al.*[1]
2138	v C-O	$CO_{(gas)}$	Figueiredo *et. al.*[1]
1680	v C=O	CO_3^{2-}	Figueiredo *et. al.*[1]
1666	v C=O	CO_3^{2-}	Figueiredo *et. al.*[1]
1608	v C=O	HCO_3^-	Figueiredo *et. al.*[1]
1487	δ CH_3	CH_3CONH_2	Foley *et. al.*[2]
1390	v C-O	HCO_3^-	Figueiredo *et. al.*[1]
1365	v C-O	CO_3^{2-}	Figueiredo *et. al.*[1]
1340	v C=O	CO_3^{2-}	Figueiredo *et. al.*[1]
1105	v BF_4	BF_4^-	Figueiredo *et. al.*[1]
1066	v BF_4	BF_4^-	Figueiredo *et. al.*[1]

References

(1) Figueiredo, M. C.; Ledezma-Yanez, I.; Koper, M. T. M. In Situ Spectroscopic Study of CO_2 Electroreduction at Copper Electrodes in Acetonitrile. *ACS Catal.* **2016**, *6* (4), 2382–2392.

(2) Foley, J. K.; Korzeniewski, C.; Pons, S. Anodic and Cathodic Reactions in Acetonitrile/Tetra-n-Butylammonium Tetrafluoroborate: An Electrochemical and Infrared Spectroelectrochemical Study. *Can J. Chem.* **2011**, *66*, 201–206.

(3) Moradzaman, M.; Mul, G. Infrared Analysis of Interfacial Phenomena during Electrochemical Reduction of CO_2 over Polycrystalline Copper Electrodes. *ACS Catal.* **2020**, *10* (15), 8049–8057.
